# Comparative Gene Expression and Physiological Analyses Reveal Molecular Mechanisms in Wound-Induced Spore Formation in the Edible Seaweed Nori

**DOI:** 10.3389/fpls.2022.840439

**Published:** 2022-03-17

**Authors:** Xiaowei Guan, Yunxiang Mao, John W. Stiller, Shanshan Shu, Ying Pang, Weihua Qu, Zehao Zhang, Fugeng Tang, Huijuan Qian, Rui Chen, Bin Sun, Du Guoying, Zhaolan Mo, Fanna Kong, Xianghai Tang, Dongmei Wang

**Affiliations:** ^1^Key Laboratory of Marine Genetics and Breeding (OUC), Ministry of Education, Qingdao, China; ^2^College of Marine Life Sciences, Ocean University of China, Qingdao, China; ^3^Key Laboratory of Utilization and Conservation for Tropical Marine Bioresources (Hainan Tropical Ocean University), Ministry of Education, Sanya, China; ^4^Department of Biology, East Carolina University, Greenville, NC, United States

**Keywords:** calcium signaling pathway, cell division, ROS, *Neopyropia yezoensis*, wound-induced spores, cell reprogramming, nori, spore-specific gene marker

## Abstract

Genetic reprogramming of differentiated cells is studied broadly in multicellular Viridiplantae as an adaptation to herbivory or damage; however, mechanisms underlying cell development and redifferentiation are largely unknown in red algae, their nearest multicellular relatives. Here we investgate cell reprogramming in the widely cultivated, edible seaweed *Neopyropia yezoesis* (“nori”), where vegetative cells in wounded blades differentiate and release as large numbers of asexual spores. Based upon physiological changes and transcriptomic dynamics after wound stress in *N. yezoensis* and its congener *Neoporphyra haitanensis*, another cultivar that does not differentiate spores after wounding, we propose a three-phase model of wound-induced spore development in *N. yezoensis*. In Phase I, propagation of ROS by RBOH and SOD elicites systematic transduction of the wound signal, while Ca^2+^ dependent signaling induces cell reprogramming. In Phase II, a TOR signaling pathway and regulation of cyclin and CDK genes result in cell divisions that spread inward from the wound edge. Once sporangia form, Phase III involves expression of proteins required for spore maturation and cell wall softening. Our analyses not only provide the first model for core molecular processes controlling cellular reprogramming in rhodophytes, but also have practical implications for achieving greater control over seeding in commercial nori farming.

## Introduction

Under environmental stress or appropriate inductive conditions, some differentiated cells can reprogram into “stem cells” that give rise to more specialized cells, or even regenerate a whole organism (Ikeuchi et al., 2016). The reprogramming of differentiated cells and acquisition of competence is widely studied in both animals and plants. In land plants, mechanical damage or herbivory can induce cell reprogramming of vegetative cells and thereby elicit tissue repair or organ regeneration (Vega-Munoz et al., 2020). Many pathways and regulatory mechanisms control these wound-induced fate transitions, including Ca^2+^ signaling, reactive oxygen species (ROS), and changes in membrane potential that perceive local wounding and convey signals that elicit long-range responses (Hilleary and Gilroy, 2018; Méndez-Hernández et al., 2019; Wasternack, 2019; Zhang et al., 2019; Ikeuchi et al., 2020). Cell reprogramming is accompanied by passage from a non-proliferative state into S phase of the cell cycle. The concomitant regulation of cyclin-dependent kinase A (CDKA) coordinates cell cycle progression with other cellular changes during reprogramming of leafy cells in the moss *Physcomitrium* (Ishikawa et al., 2011).

Molecular mechanisms for cell reprogramming have been investigated broadly in the Viridiplantae (green algae and plants) but little is known from their most closely related multicellular lineage, the Rhodophyta (red algae). Along with glaucophyte algae, these groups comprise the Archaeplastida, the primary photosynthetic lineage where chloroplasts first evolved from cyanobacterial endosymbionts about 1.5 Gya (Douzery et al., 2004; Yoon et al., 2004). Multicellular red algae are, themselves, over 1 Gy old and have acquired complex life histories and unique adaptive strategies to cope with environmental stresses. The latter is particularly true of bangiophytes inhabiting the marine intertidal, one of the most dynamic environments on the planet (Brawley et al., 2017). Transcriptomic analyses in two *Porphyra* species identified some conserved developmental regulators known from model eukaryotes, but also suggested altered roles for many key proteins and that novel mechanisms are yet to be discovered (Stiller et al., 2012). Despite these intriguing first insights, the molecular bases of cell reprogramming in red algae and their evolutionary conservation with or divergence from processes in green plants remain virtually unexplored experimentally.

Here we detail cellular reprogramming in *Neopyropia yezoensis* (Rhodophyta, Bangiales, previously named as *Pyropia yezoensis*) (Yang et al., 2020), the most cultivated and commercially valued species of edible “nori.” *Neopyropia* species have a life cycle that alternates between a leafy gametophyte “blade” and a microscopic, filamentous sporophyte. The blade is a single layer of isodiametric vegetative cells with thousands of elongated rhizoid cells in the holdfast. In another bangiophyte, *Porphyra umbilicalis*, four distinct developmental regions have been characterized in asexually reproducing blades: rhizoids, vegetative cells, differentiating neutral sporangia along blade margins, and mature neutral spores (Royer et al., 2018). Within neutral sporangia, previously vegetative cells divide repeatedly and redifferentiate into packets of neutral spores that can germinate into entirely new blades. In *N. yezoensis* sporangial packets do not form but marginal vegetative cells can redifferentiate into asexual archeospores, especially when environmental stress or exogenous stimuli are encountered (Takahashi and Mikami, 2017). Spore formation in both species involves a switch in cell fate from differentiated vegetative cells to spores that can develop into individual blades; however, because normal spore development occurs randomly, it is difficult to distinguish transforming vegetative cells until after they acquire spore-specific characteristics.

Previously, researchers found that *N. yezoensis* blades cut into small fragments of 30–50 cells can proliferate through cell division and fully redifferentiate into spores that can subsequently develop into individual blades in 5 days (Hafting, 1999; Chen et al., 2019; Suda and Mikami, 2020). With recent genomic advances in *N. yezoensis* (Cao et al., 2020; Wang et al., 2020), this fate transition from vegetative cells to spores provides an ideal research model to study the molecular mechanisms involved in cell reprogramming in red algae. In this study, we tracked transcriptional dynamics through a time series after wound stress in both *N. yezoensis* and *Neoporphyra haitanensis* (previously named *Pyropia haitanensis*), another cultivar that does not differentiate spores after wounding. Based on comparative analyses of physiological changes and transcriptional regulation after wound stress, we reveal the first landscape of regulatory mechanisms in wound-induced spore formation in *Neopyropia*.

## Materials and Methods

### *Neopyropia* Culture

*Neopyropia yezoensis* pure line RZ and *Neoporphyra haitanensis* pure line PH40 (female) were cultured in Provasoli Enriched Seawater (PES) medium (Provasoli, 1968), under 50 μmol photons m^–2^ s^–1^ with a 12 h light/12 h dark photoperiod. RZ was cultured at 10°C and PH40 at 20°C (Cao et al., 2020; Wang et al., 2020). PES medium was refreshed every 3 days.

### Transcriptome Data Collection

Using *Neopyropia* thalli 35–40 days old, basal and edge regions were removed and middle regions (defined as “pre-excised thalli” in subsequent text) were cut into small fragments (containing 30–50 cells on average) on a glass slide using a scalpel. Fragments were pipetted into conical flasks containing 250 mL PES medium and cultured in a shaker under controlled conditions [irradiance: 50 μmol photons m^–2^ s^–1^; photoperiod: 12:2 (L:D); speed:120 rpm, temperature: 10°C for *N. yezoensis* and 20°C for *N. haitanensis*]. Three culture replicates were sampled at the 6th hour, 1, 2, 3, and 5 days after wounding by collection and centrifugation. Triplicates of “pre-excised thalli” were collected as controls.

Total RNA was extracted from cut thalli fragments at all five time points and from intact thalli using the Plant RNA Kit (Omega Bio-Tek, United States). Total RNA concentrations and quality were determined with NanoDrop and agilent 2100 bioanalyzer. Messenger RNAs were captured on oligo(dT) magnetic beads to construct RNA libraries following the protocol of NEBNext^®^ Ultra™ RNA Library Prep Kit for Illumina^®^ (NEB_New England Biolabs, United States). The prepared libraries were sequenced on Illumina Hi-Seq platform using the PE150 mode.

### Transcriptome Data Analysis

After removing low-quality reads and library adapters by Trimmomatic (Bolger et al., 2014), clean reads were aligned to *N. yezoensis* and *N. haitanensis* genomes, respectively, using Hisat2 (v2.0.5) (Kim et al., 2019). We used featureCounts v1.5.0-p3 (Liao et al., 2014) to count mapped read pairs for each gene and expression levels were quantified as FPKM (Fragments Per Kilobase of transcript sequence per Millions base pairs sequenced). Differential expression analysis was performed using the DESeq2 R package (1.16.1) (Varet et al., 2016), with resulting *P*-values controlled for false discovery rate. Genes were considered differentially expressed genes (DEGs) if they had a *P*-value adjusted using the Benjamini and Hochberg’s approach was < 0.05 and fold change ≥ 2 (log_2_ (foldchange) ≥ 1 or ≤ −1). Genes were defined as spore-specific markers if they had a FPKM of ≥ 10 at the 5th day and FPKM ≤ 5 in all other stages and a fold change of FPKM of > 32 at the 5th day compared to unwounded control. We used default software parameters unless noted otherwise.

Transcriptional changes were verified using qRT-PCR. Excised fragments of *N. yezoensis* were collected at the same time points as for transcriptome analyses. Ct values were determined for triplicate technical experiments performed on triplicate biological duplications (*n* = 3). Relative fold differences were calculated based on the ΔCt method using ubiquitin C as an internal standard. Primer pairs used for qRT-PCR analyses are listed in Supplementary Table 3.

To determine differences in gene expression between *N. yezoensis* and *N. haitanensis*, we identified homologous genes in the two species through reciprocal best BLAST matches (RBH) (Lee et al., 2019), and compared transcriptional patterns between RBH pairs.

### Cell Wall Staining

Excised thallus fragments were cultured and sampled as above, placed in 25 μL culture medium, mixed with 25 μL Calcofluor (Sigma-Aldrich), incubated for 1 min in the dark, and observed under a fluorescent microscope (Nikon ECLIPSE 80i) with ultraviolet illumination.

### 5-Ethynyl-2-Deoxyuridine Labeling

The thymidine analog 5-ethynyl-2-deoxyuridine (EdU) was incorporated into chromosomal DNA during S-phase, facilitating visualization of cell cycle progression. We used TransDetect^®^ EdU Imaging Kit-488 Fluorophore (TransGen Biotech, Beijing) to label S phase cells. At time zero, 1, 2, and 3 days after wounding, EdU was added to the culture medium at a final concentration of 20 μM and incubated at 15°C for 24 h. Labeled fragments were washed three times in PBS solution. Cell were fixed in formaldehyde, stained following manufacturer instructions, and under a fluorescent microscope with 488 nm excitation illumination.

### Investigation of the Function of Ca^2+^ Channels

The effects of the glutamate receptor (GLR) agonist L-glutamate and non-specific calcium channel blocker La^3+^ (Meyerhoff et al., 2005) on cell reprogramming were examined in *N. yezoensis*. For La^3+^ inhibitory experiments, three thalli were chopped as described above and 90 total fragments (30 from each thallus) were added to a 96-well plate with PES medium. Another two sets of 90 fragments were added to PES medium with 0.5 mM LaCl_3_ (HEOWNS, 10099-58-8) and 1.0 mM LaCl_3_, respectively. Each thallus fragment was observed and imaged microscopically (OLYMPUS CKX41). The number of fragments releasing spores were counted daily. With L-glutamate treatment, each set of 30 fragments were added to 96-well plates pre-filled with PES medium (control) or PES medium with 0.1, 0.5, 1.0, or 2.0 mM L-glutamate (nacalai tespue, M2B3809). Three replicates were performed for each treatment, with numbers of fragments releasing spores counted daily. Total numbers of spores released by each fragment were counted on the 10th day after wounding.

### Reactive Oxygen Species Detection

We used the DCF method to monitor intracellular ROS levels of intact thalli and fragments after excision. Dichlorofluorescein diacetate (DCFH-DA) enters cells and is hydrolyzed by esterases, followed by oxidization by ROS to form dichlorofluorescein (DCF). Intact *N. yezoensis* thalli of equal mass (0.01 g) were cultured in 1.5 mL PES medium at 10°C. 5 μL of 5 mM DCFH-DA(Solarbio, D6470) was added and the culture incubated for 1 h. After two washes in seawater, thalli were collected through centrifugation at 1,500 rpm for 5 min, ground in liquid nitrogen, resuspended in 300 μL of 40 mM Tris-HCl buffer (pH 7.0), and centrifuged at 15,000 g for 25 min. 200 μL of supernatant was transferred to a 96-well plate. Fluorescence values of DCF were detected with Fluoroskan FL (Thermo Fisher Scientific, 5210450, excitation wavelength of 488 nm and emission wavelength at 525 nm). ROS levels of were calculated using a standard curve prepared by gradient dilution of DCF from 0 to 0.08 ng/μL (Macklin, D909945). The same procedures were applied to thallus fragments of equal fresh weight at the 1st and 5th hour after wounding. Because initial incubation with DCFH-DA took 1 h, the results presented are for fragments at the 2nd and 6th hours after wounding. The ROS detection method for *N. haitanensis* was the same as for *N. yezoensis*, except incubation was at 20°C. Three replicates were done for all treatments.

### Rapamycin Treatment

For rapamycin treatment, 1.0 mM stock solution of rapamycin was prepared in DMSO. Cut fragments of *N. yezoensis* and *N. haitanensis* were added to 96-well plates pre-filled with PES medium (control) or PES medium with 10 μM rapamycin. Morphological observations were done twice daily after wounding.

### Data Availability

The datasets generated and analyzed during the current study are available from the corresponding author upon request. The transcriptome sequencing data were deposited in NCBI under BioProject PRJNA718699, with accession numbers as SRR18559308–SRR18559325 for *N. yezoensis*, SRR18559326–SRR18559343 for *N. haitanensis*.

## Results

### Morphological Changes and Global Transcriptional Variation in Response to Wounding in *Neopyropia yezoensis*

**FIGURE 1:**
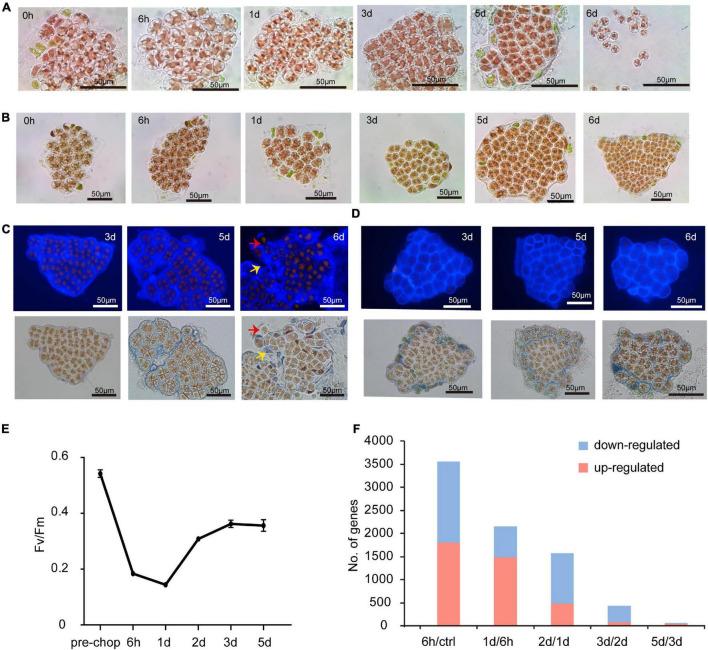
Morphological changes of cut fragments during the wound-induced spore formation and statistics of DEGs identified in the two Pyropia spices. **(A)** Cellular changes in cut fragments along the time course after wounding in *N. yezoensis*. Time refers to the duration (in hours and days) since the onset of wounding. **(B)** Cellular changes in cut fragments along the time course after wounding in *N. haitanensis.* **(C)** Calcofluor white staining of cell wall changes during the formation of sporangia and release of spores in *N. yezoensis*. The red arrow indicates a released spore without any cell wall. The yellow arrow indicates the remaining cell wall debris and interval matrix after spores released. **(D)** Calcofluor white staining of cell wall changes in *N. haitanensis.* **(E)** The variation in photosynthetic efficiency of cut fragments over time in *N. haitanensis*. Mean values of Fv/Fm were plotted with the vertical bars representing SD (*n* = 5 biological independent samples). **(F)** Histogram showing the number of up- and down-regulated genes at each time point compared to the previous time point in *N. haitanensis*.

On Day 1 (24 h after excision of blade fragments) visual inspection showed that cells rounded up and the normally stellate chloroplasts became compact. On day 2 (48 h) most cells had divided into two daughter cells and some had undergone a second division (Figure 1A). Calcofluor white staining showed the daughter cells lacked cell walls, grouped together as protoplasts and remained surrounded by the parental cell wall (Figure 1C). Both the cell wall and intervening space between cell “packets” became thicker and denser. This “packet” structure differs from typical archeosporangia in *N. yezoensis*, which contain only one differentiated spore (Nelson et al., 1999; Gao et al., 2011), and more like the neutral sporangia in *Porphyra umbilicalis* (Nelson et al., 1999; Royer et al., 2018; Figure 1A). Without clear gene markers to distinguish spore types, we use the terms wound-induced spores (WIS) in this study.

To understand regulatory mechanisms underlying the transition from vegetative cells to WIS, we profiled transcriptomic data for intact thalli (after removal of rhizoid regions) and excised fragments across five time points (see section “Materials and Methods”). More than 3,000 differentially expressed genes (DEGs) encoding diverse functions were found at each timepoint compared to the intact control, with 1,292 common to all time points (Supplementary Figures 1, 2). Numerous DEGs occurred specifically at each different timepoint (Supplementary Figure 1), indicating potential links among their functions and sequential cellular changes through time. Because transcription factors (TFs) regulate gene expression and developmental transitions, variations in TF expression are likely important for understanding cell status and fate. We identified a number of differentially expressed TFs, but only six were shared across all timepoints (Supplementary Figure 3). Interestingly, the number of temporally specific TFs at 6 h (22), day 2 (16), and day 5 (33) were substantially higher than on day 1 (0) and day 3 (1). Combined with morphological changes observed during wound-induced spore formation, TF expression suggests that significant physiological and molecular changes occur in three distinct stages.

### Morphological Changes and Global Transcriptional Variation in Response to Wounding in *Neoporphyra haitanensis*

As in *N. yezoensis*, cells in excised fragments from *Neoporphyra haitanensis* initially became round with compacted plastids, surrounding cell debris was eliminated (Figure 1B) and thicker cell walls appeared (Figure 1D); however, fragments then regenerated individual thalli rather than developing sporangium-like structures and some fragments initiated rhizoids at their boundaries. Photosynthetic efficiency (Fv/Fm) in *N. haitanensis* fragments declined immediately after wounding, reaching its lowest level at day 1, then increased on days 2 and 3 and appeared stable through day 5 (Figure 1E). Morphological and physiological changes in *N. haitanensis* indicate rapid tissue repair over the first 2 days, after which fragments grow into individual thalli without any obvious cell fate transitions.

The distinctive developmental fates after wounding between the two species indicate different transcriptional responses. Therefore, we collected transcriptome data from *N. haitanensis* using the same protocol as from *N. yezoensis*. In sample clustering based upon temporal dynamics of transcriptional levels, the pre-wound control, hour 6 and day 1 each formed distinct clusters, while the other three samples were less distinguishable (Supplementary Figure 4). Comparing expression at each time point to the previous stage found 3,566 DEGs at hour 6 compared to unwounded cells, decreasing to 1,574 DEGs by day 2 and declining to almost no change between days 3 and 5 (Figure 1F). The transcriptional dynamics of 25 photosynthesis-related DEG genes showed mostly very high absolute transcript abundance (FPKM > 1,000) in control thalli, with progressive down-regulation in the first day after wounding. Expression began to rise on day 2 and continued thereafter, approaching control levels by day 5 (Supplementary Figure 5), consistent with a recovery of photosynthesis efficiency after an initial stress response to wounding. Therefore, patterns of transcriptional regulation, both global and of photosynthetic genes, correlate with morphological changes and photosynthetic efficiency; all are consistent with our hypothesis that a wound response involving complete tissue repair is complete in 2 days in *N. haitanensis*. Because the response in *N. yezoensis* involves rapid changes in cell fate, we further focused on a comparison of transcriptional differences between *N. haitanensis* and *N. yezoensis* at early time points following wounding.

### Reactive Oxygen Species-Mediated Systematic Signaling Was Involved in Wound Response in *Neopyropia yezoensis*

**FIGURE 2:**
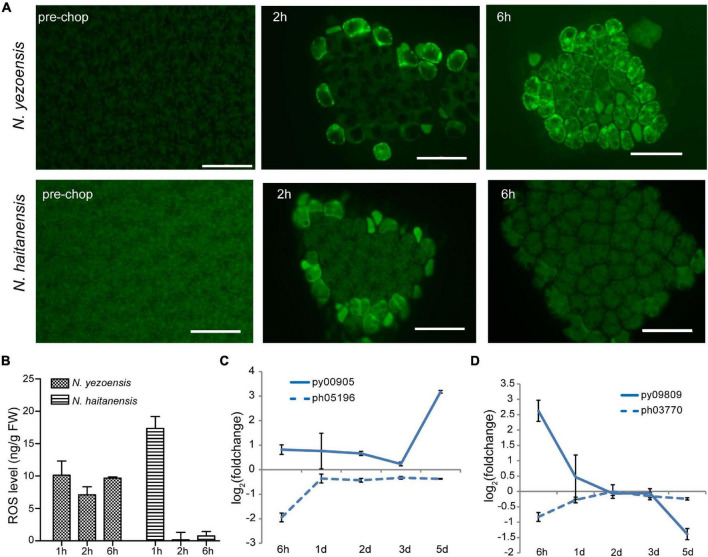
ROS-mediated systematic signaling in *N. yezoensis* thallus fragments in response to wound stress. **(A)** ROS staining of intact thalli and fragments 2 and 6 h after wounding. Upper panel images are *N. yezoensis*; lower *N. haitanensis*. Dark green backgrounds in each image are auto-fluorescence. Bars represent 50 μm in each panel. **(B)** Total ROS detected in excised fragments in the two species. Columns and vertical bars represent the average values and SD (*n* = 3). **(C)** Transcriptional variation of RBOH genes in *Neopyropia* after wounding. The log_2_ value of FPKM foldchange compared to the intact sample for each time point was used for plotting. The solid line and dashed line represent genes from *N. yezoensis* and *N. haitanensis*, respectively. The same line colors indicate the two genes are best reciprocal hits. **(D)** Transcriptional variation of SOD genes in *Pyropia* after wounding.

ROS, mainly superoxide anion (O_2_^–^) and H_2_O_2_, are important wound signals in plants (Hilleary and Gilroy, 2018). ROS were observed in cells facing cut edges by 2 h post-wounding in both species. Initially localized near the plasma membrane of edge cells, the signal spread internally in *N. yezoensis*; at 6 h ROS were detected in most cells (Figure 2A). The gradual propagation of a signal from wound sites to other intact cells indicates continuous ROS generation and transport in *N. yezoensis*. In *N. haitanensis*, however, a ROS signal was strong initially, but remained localized outermost cells and disappeared by 6 h post-wounding (Figure 2A). Quantitation of total ROS at corresponding time points in the two species confirm differences observed microscopically (Figure 2B).

In vascular bundles of *Arabidopsis* responding to environmental stress, respiratory burst oxidase proteins (RBOHD and RBOHF) produce O_2_^–^ through transfer of electrons from NADPH to molecular oxygen, propagating both local and systemic ROS signaling (Miller et al., 2009; Zandalinas et al., 2020). The O_2_^–^ then can be converted to H_2_O_2_ by SOD catalysis or natural disproportionation. Apoplastic production of H_2_O_2_ subsequently is transported across the plasma membrane and mediates systematic signaling in stress acclimation. We identified eight RBOHs in both *N. yezoensis* and *N. haitanensis*; seven have multiple transmembrane helices suggesting localization to the plasma membrane. Because propagation of ROS occurs within 1 day, we compared transcriptional levels of RBOHs at 6 h in the two species. In *N. yezoensis*, py00905 showed elevated transcription by 1.8-fold, while its *N. haitanensis* ortholog, ph05196, was significantly down-regulated (FPKM from 9.2 to 2.4, Figure 2C and Supplementary Figure 6A). The other two *N. yezoensis* RBOH genes, py02900 and py04018 exhibited significant and transient up-regulation at 6 h, while their counterparts in *N. haitanensis* were also induced, albeit to a lower degree by 1.6- and 3.1-fold, respectively (Supplementary Figure 6B, as verified by qRT-PCR in Supplementary Figure 7).

Among the 11 SOD genes found in *N. yezoensis*, transcription of only one (py09809) was sharply up-regulated (FPKM from 116 to 741) at 6 h post-wounding (Figure 2D as verified by qRT-PCR in Supplementary Figure 7); other pySODs were down-regulated, as were all *N. haitanensis* SOD homologs (Supplementary Figures 6C,D). Therefore, the increased expression of these RBOH and SOD genes in *N. yezoensis* appears related to the propagation of an ROS wave during the initial response to wound stress. Aquaporin (AQP) biomembrane channels are essential for transporting water, H_2_O_2_, and other small molecules and function in cytoplasmic import of apoplastic H_2_O_2_ induced by herbivory or pathogenic infection (Tian et al., 2016). We found four AQP genes in *N. yezoensis*; two were up-regulated along the full time course and the other two down-regulated. Similar transcriptional changes were observed in the four *N. haitanensis* homologs (Supplementary Figure 6E).

### Calcium Signaling Pathway

**FIGURE 3:**
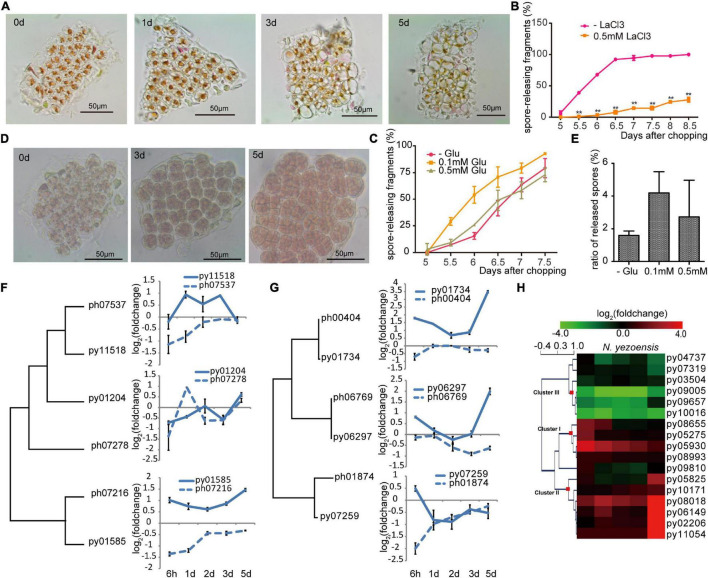
Ca^2+^ signaling in the wound-induced spore formation in *N. yezoensis*. **(A)** Effects of 1.0 mM LaCl_3_ on boundary repair and sporangia formation, causing eventual cell death. Morphological changes in the same cut fragment tracked at 0, 1, 3, and 5 days after wounding. **(B)** Effect of 0.5 mM LaCl_3_ on the percentage of cut fragments able to release spores. Average percentages of spore-releasing fragments plotted over time in control and LaCl_3_-treated media; error bars represent the SD (*n* = 3, 30 fragments/replicate as described in “Materials and Methods” section). **P < 0.01. **(C)** The percentage of cut fragments releasing spores with different dosages of L-glutamate. The plot follows the description for **(B)**. **(D)** Morphological changes in a cut fragment treated with 0.1 mM L-glutamate. **(E)** Effect of 0.1 mM of L-glutamate on spore release. Columns represent the ratio of released spores to original cells in corresponding fragment with the SD shown (*n* = 3, 30 fragments/replicate). **(F)** Transcriptional variation of *N. yezoensis* GLR genes and their *N. haitanensis* counterparts. The dendrogram was constructed by maximum-likelihood and plots for each gene were done as described in Figure 2C. **(G)** Transcriptional variation of *N. yezoensis* calmodulin genes and their *N. haitanensis* counterparts. The dendrogram and plots are as described in **(F)**. **(H)** Heatmap showing the transcriptional variation of CDPK genes in *N. yezoensis* and *N. haitanensis*. The log_2_ value of FPKM foldchange compared to the intact sample at each time point (see “Materials and Methods” section) for individual genes are displayed, as indicated by the color bar. The three clusters of CDPK genes discussed in the text were generated by Hierarchical Clustering and are indicated by red squares on the nodes.

In plants, changes in cytosolic Ca^2+^ concentration ([Ca^2+^]_*cyt*_) following wounding contribute to local and systemic signaling that primes non-damaged regions to mount defenses (Hilleary and Gilroy, 2018). Ca^2+^ signaling was previously reported to be required in the early development of archeospores in *N. yezoensis* (Li et al., 2009; Takahashi et al., 2010). To test whether Ca^2+^ signaling is involved in WIS development in *N. yezoensis*, we treated excised fragments of *N. yezoensis* and *N. haitanensis* with 1.0 mM LaCl_3_, a non-specific calcium channel blocker. Cut edges from both species still had attached cellular debris at day 5 after wounding and cells gradually died within a week (Figure 3A). When treated with a lower concentration of LaCl_3_ (0.5 mM), however, cells remained alive but typical sporangial packets did not form in most *N. yezoensis* fragments; the percentage of fragments releasing spores dropped to less than 20% of normal (Figure 3B). Therefore, blocking calcium transport in *N. yezoensis* attenuates wound repair and inhibits the transition of vegetative cells into spores, ultimately leading to widespread cell death. Based on these results, calcium signaling appears to play essential roles in eliciting a wound response in both *Pyropia* species, but also in cell fate transition after wounding in *N. yezoensis*.

Long-distance transport of a Ca^2+^ signal relies on a family of cation-permeable ion channels, the glutamate receptor-like (GLR) proteins (Meyerhoff et al., 2005). There are three genes encoding GLRs in each *Pyropia* genome. In *N. yezoensis*, expression of py01585 was sharply elevated at 6 h and remained so through day 5 (as verified by qRT-PCR in Supplementary Figure 7). Transcription of Py11518, although barely changed initially, increased significantly from the 1st to 3rd day, then dropped back to control level by day 5. Homologs of these two genes in *N. haitanensis* showed similar transcriptional patterns to each other, but not to *N. yezoensis* orthologs, with continuous down-regulation during the first 2 days and then a return to near control levels afterward. Py01204 was slightly down-regulated at the onset of wound stress, whereas a third *N. haitanensis* homolog ph07278 showed transient up-regulation on day1 (these two sequences are not clearly orthologous in a phylogenetic analysis) (Figure 3F and Supplementary Figure 8).

We added extracellular L-glutamate (agonist of GLR; Toyota et al., 2018) to *N. yezoensis* fragments to study the effect of triggered glutamate receptors in wound defense and cell reprogramming. Treatment with 0.1 mM of L-glutamate resulted in spore release from > 25% of excised fragments 5.5 days after wounding. By day 6, this increased to more than 50% compared to only 13% in untreated samples (Figure 3C). Although only moderate quantitative differences were observed between Glu-treated and control samples, glutamate does appear to accelerate the formation and release of spores. More importantly, we observed crowded sporangia containing increased number of pre-spores in 0.1 mM Glu-treated fragments on day 5 (Figure 3D). Total numbers of spores released per fragment also increased. The ratio of spores to original cells was around 2 in untreated samples, but more than 4 in treated fragments (Figure 3E). Although 0.5 mM L-glutamate treatment did not visibly impact spore formation, higher concentrations (1–2 mM) resulted in the appearance of many vacuoles that pushed pigments to the side of the cell. Finally, only 21.3% of 1.0 mM Glu-treated fragments released spores; the remaining fragments resumed regular growth afterward. Therefore, low concentrations of L-glutamate promote cell cycle progression and increase the rate of cell division, leading to a larger number of spores generated. We suggest that L-glutamate activates GLRs and promotes the transduction of calcium signaling as observed in land plants (Toyota et al., 2018).

Calmodulin proteins act as important sensors in developmental processes by undergoing conformational changes after binding Ca^2+^and regulating downstream proteins (Romeis and Herde, 2014). Previously increased calmodulin expression was found during the development of archeospores in both *N. yezoensis* and *Phycocalida chauhanii* (Song et al., 2020; Li et al., 2021). All three *N. yezoensis* calmodulin proteins displayed opposite trends in transcriptional regulation compared to their counterparts in *N. haitanensis*. In *N. yezoensis*, calmodulins are coordinately up-regulated to varied degrees at the onset of wound response, then return to control levels over the following 2 days. During later formation of sporangia, transcription of py01734 and py06297 dramatically increase again. In contrast, two calmodulin genes in *N. haitanensis* maintained basal or only slightly changed levels of transcription, and the other (ph01874) was actually down-regulated substantially at 6 h (Figure 3G and Supplementary Figure 8).

Similar differences in transcriptional dynamics between the two species were observed in the other potential Ca^2+^ sensors, the Ca^2+^-dependent protein kinases [CAMK-like and CDPK-like (11)] (Romeis and Herde, 2014). Genes for both kinase families in *N. yezoensis* underwent strong changes in expression during wound-induced cell reprogramming compared to their *N. haitanensis* homologs, which varied in ranges less than twofold (Figure 3H and Supplementary Figures 8, 9). The 15 *N. yezoensis* CDPK- and CAMK-like genes showed diverse expression patterns in response to wounding; most that had substantially altered levels of transcription could be categorized into three clusters (Figure 3H). Transcription of three genes in cluster I was upregulated at 6 h, followed by a downward trend back to basal levels (as verified by qRT-PCR in Supplementary Figure 7). Five genes (cluster II) barely changed expression at the onset, but had an explosive increase on day 5. Given that spores are maturing on the 5th day, the dramatic up-regulation of these protein kinases likely contributes to physiological activities associated with the late maturation and release of spores.

Genes in cluster III exhibited the highest absolute transcript levels in control thalli and were significantly repressed after wounding, although two returned to near pre-wounding levels by day 5. Their high expression levels in undamaged thalli and down-regulation after stress suggest these kinases could be responsible for signal transduction during regular, vegetative growth, and must be down-regulated during developmental changes leading to spore production. This is further supported by the lack of significant expression changes of orthologs in *P. haitenensis*, where cell growth continues after wounding without a change in cell fate. Several other related kinases do not appear to be involved in this stress response based on a lack of change in expression following wounding (Figure 3H and Supplementary Figure 9).

### Cell Cycle Progression During Cell Reprogramming in *Neopyropia yezoensis*

**FIGURE 4:**
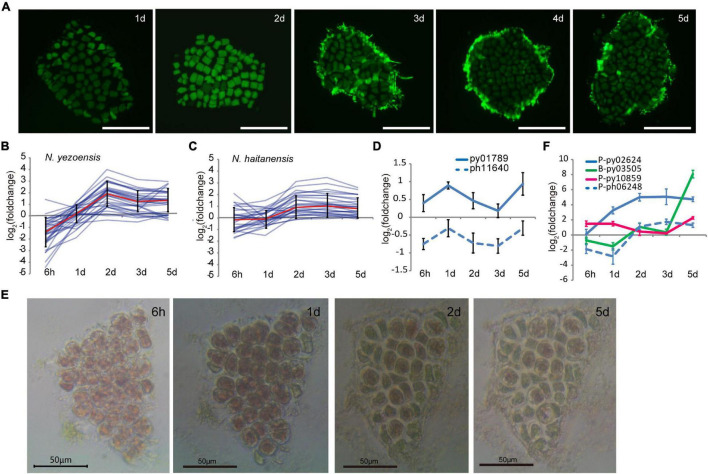
Cell cycle progression during wound-induced spore formation. **(A)** S-phase cells stained by EdU in the cut fragments along the time course. Fluorescence at the edge of fragments on 3–5th days are remaining reagents. Bars represent 50 μm in each panel. **(B)** Transcriptional variations of DNA-replication related genes in *N. yezoensis*. The red line represents the mean values of log_2_ (foldchange) of all the related genes, and the bars indicate standard errors. **(C)** Same plot as 4C but for *N. haitanensis*. **(D)** Transcriptional variation of TOR genes in the two *Pyropia* species. **(E)** Cellular changes in a *N. yezoensis* thallus fragment treated with rapamycin. **(F)** Transcriptional variation of three cyclin genes in *N. yezoensis*. The dashed line in blue was the *N. haitanensis* ortholog of py02624. The plots in **(D,F)** were done as described in Figure 2C.

In plants, reprogramming of differentiated somatic cells into cells with increased developmental plasticity is accompanied by reentry into the cell cycle from a non-proliferative state to S phase (Boer and Murray, 2000). The release of many more spores than the number of cells originally present in excised fragments indicated that cell proliferation occurs after wounding in *N. yezoensis*; however, the timing of cell divisions was unclear. We used EdU to identify cells undergoing active DNA replication. Because cells must be incubated with EdU for at least 24 h, our earliest results are for 1 day after wounding. At day 1, EdU was incorporated in nuclei of boundary cells in cut fragments, indicating cells nearest the wound were the first to enter S phase (Figure 4A). By day 2, most cells were EdU-positive except a few relatively distant from fragment edges. By day 3, only some central cells replicating DNA, presumably those that had lacked EdU incorporation on day 2. By days 4 and 5, S-phase cells were barely detected and spore release began in distal regions. These observations reveal a timeline of cell cycle progression extending from the “outside to inside” of fragments; edge cells are induced into S phase soon after wounding and a signal spreads rapidly throughout the fragment by day 2. After this wave of intensive cell divisions, mature spores appear to arrest in a G_0_ state until germinating after release.

We analyzed transcription of 35 genes annotated as involved in DNA replication, including DNA polymerase subunits, licensing factors, DNA ligase, replication factors, and ribonuclease H1. Most displayed synchronous changes in transcription (Figure 4B, several were verified by qRT-PCR in Supplementary Figure 7), with transcript levels decreasing to varied degrees by hour, then rebounding to a peak on day 2. Although expression dropped somewhat afterward, levels remained higher than in unwounded thalli. Genes related to mismatch and base excision repair exhibited similar trends in transcriptional variation (Supplementary Figure 10). The synchronous co-expression of DNA synthesis-related genes is consistent with the timing of DNA replication revealed by EdU labeling. DNA-synthesis genes in *N. haitanensis* displayed gentler changes in expression during the first 2 days following wounding, consistent with the lack of spore proliferation in that species (Figure 4C). Except for three that increased more than twofold, only slight changes in transcription occurred by hour 6 and day 1. By day 2, small increases in transcription were observed, indicating a recovery of vegatative growth after wounding.

The target of rapamycin (TOR) signaling pathway promotes growth by regulating the cell cycle in response to mitogenic signals in diverse eukaryotes (Li et al., 2017; Ahmad et al., 2019). Transcription of the TOR gene in *N. yezoensis* was elevated slightly at hour 6 and then substantially on day 1, whereas its counterpart in *N. haitanensis* was continuously down-regulated after wounding (Figure 4D). With rapamycin inhibition of TOR, cells in *N. yezoensis* became round with enlarged intercellular spaces and cell division did not occur. Cells at fragment edges died by day 2, followed by those toward the middle (Figure 4E). The same treatment in *N. haitanensis* did not kill cells (Supplementary Figure 11). The complete block of cell proliferation by rapamycin and subsequent cell death only in *N. yezoensis* indicate that TOR signaling is essential for promoting cell cycle progression in wound-response developmental reprogramming. In green plants, TOR and downstream signaling mechanisms regulate two complexes; D-type cyclins (CYCD)/A-type cyclin-dependent kinase (CDKA) and CYCB/CDKB, which control G1/S and G2/M transitions, respectively (Meijer and Murray, 2000; Vandepoele et al., 2002; Ahmad et al., 2019). Seventeen genes encode cyclin proteins in the *N. yezoensis* genome, most with respective homologies to A-type (1), B-type (2), H/T/L-type (3), and U/P-type (11) cyclins (Supplementary Figure 12). The other three are not clearly identifiable to plant cyclin types and reciprocal best matches were not found in the other red algal genomes; thus, we refer to them as *Pyropia*-specific cyclins. Interestingly, D-type cyclins appear to be absent from *Pyropia*, as reported for other red algal genomes (Brawley et al., 2017).

To identify cyclins potentially involved in cell cycle progression, we compared transcriptional dynamics of *N. yezoensis* cyclin genes to their *N. haitanensis* orthologs. Transcriptional patterns were diverse and not relatable to family type, reflecting different roles in the regulation of phase transition. Interestingly, we found no orthologs of two cyclins (P-type py10859 and B-type py03505) among *N. haitanensis* sequences. Moreover, transcripts of py03505 were barely detected in control thalli and accumulated almost exclusively on days 1 and 2nd post-wounding. Expression of P-type py10859 increased significantly at hour 6 and day 1, declined on days 2 and 3, then increased again on day 5 (Figure 4F). The unique presence and elevated expression of these two cyclins during the 2 days after wounding strongly suggest they contribute to WIS cell cycle transitions specific to *N. yezoensis*.

Among the 11 CDK genes identified in *N. yezoensis*, four showed simultaneous up-regulation over the entire time course while their counterparts in *N. haitanensis* were mostly unchanged or down-regulated. For example, P-type py02624 exhibited increased transcription throughout cell reprogramming and spore development processes, while its ortholog (ph06248) was down-regulated initially, then up-regulated afterward. Py09517 was the reciprocal best match of CDKA in the unicellular red alga *Cyanidioschyzon merolae* and the latter was reported to be responsible for the G1/S Transition (Fujiwara et al., 2020). Although py09517 was highly expressed in control thalli, expression declined strongly after wounding. If the function of this CDKA in regular cell cycle progression is evolutionarily conserved in red algae, the down-regulation of py09517 after damage suggests that *N. yezoensis* cells reenter the cell cycle in wound-induced reprogramming using an independent cyclin-CDK complex.

### Structural Remodeling of the Cell Wall for Releasing Spores

**FIGURE 5:**
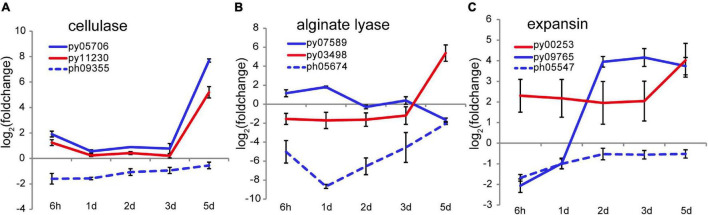
Transcriptional variation of cellulase **(A)**, alginate lyase **(B)**, and expansin **(C)** genes in *N. yezoensis* and *N. haitanensis* after wounding. The plots are as described in Figure 2C.

At 4–5 days after wounding calcofluor white staining showed pre-spores enclosed in a thick cell wall forming packet structures resembling sporangia. After spore release, cell wall debris and the polysaccharide matrix remained in the intervening spaces (Figure 1B), consistent with observations of naturally releasing archeospores in *Neopyropia* (Gao et al., 2011). Although the cell walls and matrix are not fully degraded, structural remodeling is required to facilitate spore release. Two cellulase genes, py05706 and py11230, found in *N. yezoensis* showed dramatically elevated transcription on day 5 (as verified by qRT-PCR in Supplementary Figure 7), while their counterparts in *N. haitanensis* were somewhat down-regulated over the full time course (Figure 5A). Transcriptional variation of other genes harboring a glycoside hydrolase (GH) domain were rather diverse, including the 4 and 6 genes that, respectively, encode mannosidase and galactosidase (Supplementary Figure 13), suggesting complicated cell wall changes throughout the process. Although alginate has not been reported in cell walls of *Bangiales*, we found two apparent homologs of alginate lyase genes in *N. yezoensis*. Py07589, with near sequence identity to an alginate lyase gene previously identified in *N. yezoensis* (Inoue et al., 2015), exhibited elevated transcription from the onset of wound-stress to day 1st, then was down-regulated until day 5. Its counterpart in *N. haitanensis* showed an opposite trend with significant down-regulation over the first 2 days followed by a gradual return to nearly pre-stress levels. Py03498 was downregulated initially, but showed substantially increased expression on the 5th day (Figure 5B). Interestingly, although Py03498 shows clear red algal scaffold synteny, it returns no blast matches to either the *N. haitanensis* or *P. umbilicalis* genome (Brawley et al., 2017), suggesting it is uniquely present in *N. yezoensis*, likely acquired horizontally from a bacterium.

Two genes encoding cell wall-loosening expansins were identified in *N. yezoensis*. Py00253 transcription was barely detectable on day 1 but expression increased on day 2 and remained high through day 5. In contrast, py09765 was specifically activated on day 5 (as verified by qRT-PCR in Supplementary Figure 7). Transcription of the *N. haitanensis* ortholog (ph05547) of py09765 was initially low (FPKM = 5.6) and then barely detected afterward (FPKM < 5.0) (Figure 5C). An ortholog of py00253 was not found in the *N. haitanensis* genome assembly. Synergy between expansins and cellulases was reported in enzymatic hydrolysis of cellulose in *Bacillus* (Zhang et al., 2021). Therefore, the upregulation of cellulase, expansin and, potentially, alginate lysase genes likely contribute to spore liberation by loosening cell walls and the extracellular matrix.

### Identification of Gene Markers of Wound-Induced Sporangia

On the 5th day after wounding, sporangia containing 2–4 cells become more protuberant and surrounded by rigid cell walls with larger spaces between spores. A few marginal sporangia release at this point, though most spores are discharged a day later. Therefore, it appears that vegetative cells have fully redifferentiated as spores by day 5. To identify gene markers of spore formation (see criteria in “Materials and Methods” section) we evaluated transcriptional levels of genes through the full time course in this study, as well as in the two *Neopyropia* life history stages (Wang et al., 2020). We found 108 putative sporangia-specific genes. Although 51.8% lack annotations, suggesting they could be bangiophyte-specific, diverse biological functions are encoded by identifiable spore marker genes (Supplementary Table 2 and Supplementary Figure 14). PyKNOX, a knotted-like homeobox gene and proposed marker for conchosporangia in *N. yezoensis* (Mikami et al., 2019; He et al., 2021), was predominately expressed on day 5 post-wounding; its expression jumped to FPKM > 50 after meansuring at < 1 FPKM at other time points. Transcription of its ortholog in *N. haitanensis* remained barely detectable across the full time course. This specific expression in both conchosporangia and the wound-induced sporangia points to PyKNOX as a common marker for spores that will develop into thalli.

## Discussion

**FIGURE 6:**
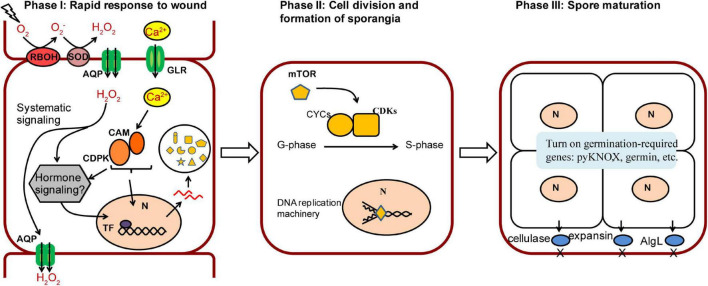
A proposed model of three phases in wound-induced cell fate reprogramming in *N. yezoensis*. Systematic signaling of ROS coupled with the Ca^2+^-dependent signaling triggers an initial response to wound stress in Phase I, including activation of CAM/CDPK for downstream transcriptional regulation and possible phytohormone-related regulation yet to be determined. The induced expression of mTOR functions as a master regulator in turning on cell cycle progression by regulating multiple cyclins and CDKs (Phase II). Once cell divisions complete, sporangia containing 2–4 pre-spores are formed. Proteins required for spore release and germination accumulate in Phase III.

Based on our results, we proposed a three-phase model of wound-induced spore development in *N. yezoensis* (Figure 6). In phase I a rapid response is triggered after wounding. Propagation of ROS by RBOH and SOD coupled with channeling to neighboring cells, elicites systematic transduction of the wound signal, and Ca^2+^ dependent signaling invokes cell reprogramming. Up-regulation of calmodulins and protein kinases likely contribute to the induction of multiple genes by activating related TFs. Although downstream components of ROS remain unclear, a phytohormone-related signaling pathway appears to be a promising candidate for induction of targeted genes (see below). Once cell fate reprogramming is determined, phase II involves cell divisions from outside to inside cells of excised thallus fragments. The TOR signaling pathway and transcriptional regulation of cyclin and CDK genes are involved in cell cycle progression. In Phase III, sporangia form and proteins required in spore maturation, germination and cell wall loosening accumulate; these include pyKNOX, an apparent common marker for multiple sporangia-types, as well as Hh-proteins and cellulases, alginate lyases, and expansins that aid in structural remodeling and loosening of cell walls. We also note that multiple genes related to ROS and Ca^2+^ signaling (e.g., *RBOH*, *GLR*, *calmodulin*) exhibit elevated transcription on day 5, in addition to their up-regulation at hour 6. Given previous results showing that Ca^2+^ influx helps to establish and maintain archeospore cell polarity (Li et al., 2009), our results indicate that ROS and Ca^2+^ pathways also are involved in spore maturation and subsequent germination.

Cell fate reprogramming of differentiated cells is a widely studied phenomenon in plants. Under appropriate inductive conditions, such as herbivory, physical damage or external chemical signals, somatic cells can dedifferentiate into a pluripotent state. Competencies for cell reprogramming differ across organisms. In vascular plants, wounded tissues often proliferate to form a callus that can then develop into shoot or meristematic tissues, whereas in ferns and bryophytes a differentiated cell can transit directly into apical stem cells (Ishikawa et al., 2011). We observed remarkable differences in cell development from excised thallus fragments of two similar red algal species. In *N. yezoensis*, vegetative cells undergo cell reprogramming and divisions to form spores, whereas *N. haitanensis* fragments experience rapid boundary repair and resume normal, vegetative growth. At least some of those differences appear attributable to genes uniquely found in *N. yezoensis*.

Although our results demonstrate similarities in wound-induced reprogramming between *N. yezoensis* and green plants, there also are key differences. In plants, CYCD and CDKA formed a complex and regulates the G1/S phase transition in cell cycle progression. Activation of CDKA and induced transcription of CYCD were observed in cell reprogramming in *Physcomitrella* (Ishikawa et al., 2011). CYCD has not been found in red algal genomes to date and a putative homolog of CDKA in *N. yezoensis* was significantly down-regulated after wounding, although its function was previously characterized in unicellular red algae *C. merolae* (Fujiwara et al., 2020). Therefore, alternative cyclin-CDK complexes are likely involved in wound-induced G1/S transitions during cell fate reprogramming in *N. yezoensis*.

As in green plants, our results show that *Pyropia* relies on ROS-mediated signaling to launch a fragment-wide response to wound stress. In *Arabidopsis*, expression of the AQP gene AtPIP1;4 is induced in pathogen-initiated H_2_O_2_ transport (Tian et al., 2016). In *Pyropia*, transport of H_2_O_2_ (the main component of ROS) throughout excised fragments could rely on biomembrane AQP channels based on up-regulation of two AQP genes after wound stress in *N. yezoensis*; however, increased AQP expression also was found in *N. haitanensis* where the ROS signal is not propagated fragment-wide. It is possible that transcriptional regulation of AQPs is generally conserved in the two species but different protein-level regulation leads to differential transport of H_2_O_2_ into neighboring cells. Alternatively, distinctive ROS signaling could result from H_2_O_2_ propagation *via* RBOH and SOD progressively induced by as yet unidentified signals in *Neopyropia*, rather than by AQP-dependent translocation. If so, the red algal response has diverged substantially from green plants where H_2_O_2_ is propagated *via* RBOH and SOD, as well as AQP in systematic ROS signaling.

Although our model explains key steps in WIS cell reprogramming and spore release in *N. yezoensis*, much remains unclear regarding links between wound signaling and down-stream physiological. In green plants, ROS and Ca^2+^ signaling result in biosynthesis and accumulation of phytohormones such as auxin and jasmonic acid (JA), which in turn induce transcription factors that promote regeneration; for example, in adventitious root formation and TOR activation in shoot apexes in *Arabidopsis* (Canher et al., 2020; Huang et al., 2020). Several phytohormones, including IAA and JA, were detected *via* mass spectrometry in *Neopyropia* thalli in both our (data not shown) and other studies (Mikami et al., 2016); however, we are unable to identify auxin biosynthesis related genes in *Neopyropia*. It is possible that these genes are present in red algae, but are so highly divergent that they are not recongnized in blast similarity searches. Alternatively, phytohormones or precursors could be provided by epiphytic bacteria and transported into *Pyropia* cells, as has been shown for IAA supplied to diatoms by Sulfitobacter (Amin et al., 2015).

Whether expressed internally or acquired from bacteria, auxin or JA could trigger expression of transcription factors, as they do with AP2/ERF transcription factor ERF115, WIND1, etc., in wound-induced organ regeneration in plants (Zhang et al., 2019; Canher et al., 2020). Homologs of these specific TFs have not been identified in *Neopyropia*; however, we found dozens of putative TFs that are up- or down-regulated at various time points after wounding. Further studies are needed to elucidate the effects of phytohormones and bacterial-algal interactions during red algal development, and to characterize TF regulatory targets and their biological functions. It’s also possible that some TFs function in wound-induced spore formation, but are not regulated at transcriptional level and, thus, not identified *via* transcriptomic analysis.

Beyond wound-induced spore formation, our findings of homologs of alginate lyase involved in spore release suggest intriguing and previously uncharacterized aspects of bangiophyte cell wall structure. Although alginates have not been reported in cell walls in the Bangiales, other complex polyuronic acids are present but tend to be degraded during biochemical processes typically used to isolate more common cell wall components (Wahlstrom et al., 2018). Alginate lyases have a variety of known substrate specificities (Kim et al., 2011) and it appears reasonable that these enzymes degrade some undetermined polysaccharide component(s) of the complex cell wall matrix present in bangiophytes. In any case, the conservation of an alginate lyase ortholog between *Pyropia* and *Porphrya*, genera that diverged over 200 MYA (Xu et al., 2018), suggests this enzyme has an important function in loosening cell walls for spore release, one highlighted for the first time in our results. Moreover, the acquisition of both *N. yezoensis* alginate lyases through HGT reinforces the dynamic genetic interplay between red algae and their surrounding biota (Wang et al., 2020). The main components of the *Neopyropia* cell wall and extracellular matrix are cellulose, beta-1,4-mannan and linear galactan polymers (porphyrans) (Mukai et al., 1981). Our findings that two cellulase genes have dramatically elevated transcription on day 5, but are down-regulated in *N. haitanensis*, suggest their important contribution to cell wall softening and remodeling in spore release.

In summary, our comprehensive demonstration of both physiological and transcriptional changes underlying spore formation provides important new insights into the molecular mechanisms of wound response and cell reprogramming in red algae, and point to exciting new directions for research into hormone-induced gene regulation. Further, we identify key genetic signatures involved in spore formation that can help pave the way toward genetic engineering of *N. yezoensis* to provide greater control over spore production in nori farming.

## Data Availability Statement

The datasets presented in this study can be found in online repositories. The names of the repository/repositories and accession number(s) can be found below: Bioproject accession number: PRJNA718699, the SRA accession numbers: SRR18559308–SRR18559325 for *N. yezoensis*, and SRR18559326–SRR18559343 for *N. haitanensis*.

## Author contributions

DW designed the research. XG, JS, YM, SS, YP, and WQ performed the research. FT, HQ, RC, and BS prepared samples. DG, ZM, FK, and XT contributed new reagents and analytic tools. DW, JS, XG, and ZZ analyzed the data. DW and JS wrote the manuscript. All authors contributed to the article and approved the submitted version.

## Conflict of Interest

The authors declare that the research was conducted in the absence of any commercial or financial relationships that could be construed as a potential conflict of interest.

## Publisher’s Note

All claims expressed in this article are solely those of the authors and do not necessarily represent those of their affiliated organizations, or those of the publisher, the editors and the reviewers. Any product that may be evaluated in this article, or claim that may be made by its manufacturer, is not guaranteed or endorsed by the publisher.
