# Supplementary material for: Comparative Gene Expression and Physiological Analyses Reveal Molecular Mechanisms in Wound-Induced Spore Formation in the Edible Seaweed Nori
Source: Front Plant Sci. 2022 Mar 17;13:840439. doi: 10.3389/fpls.2022.840439 (PMC8969420; doi:10.3389/fpls.2022.840439)
Supplement: Supplementary file 3 [file Data_Sheet_1.PDF]

Supplemental Tables and Figures

Table S1. Statistics of transcriptome data used in this study.

	sample	clean reads	uniquely aligned reads	aligned ratio	aligned genes
Neopyropia yezoensis	pyctrl-1	65866368	60665680	92.10%	11037
	pyctrl-2	51342328	47158636	91.85%	11097
	pyctrl-3	57598446	53126177	92.24%	11063
	py6h-1	55183752	50925992	92.28%	10937
	py6h-2	46819018	42992617	91.83%	11043
	py6h-3	63274676	58592506	92.60%	11084
	py1d-1	56224898	52484164	93.35%	10901
	py1d-2	59622380	55409867	92.93%	10910
	py1d-3	61216272	55109149	90.02%	10842
	py2d-1	50696756	46711005	92.14%	11091
	py2d-2	63504832	58189276	91.63%	11317
	py2d-3	70246720	64051434	91.18%	11233
	py3d-1	53710564	49099415	91.41%	11121
	py3d-2	53597532	49823005	92.96%	11168
	py3d-3	72849356	67674304	92.90%	11170
	py5d-1	63864584	58684241	91.89%	11295
	py5d-2	57001576	51863564	90.99%	11429
	py5d-3	55538604	49960945	89.96%	11293
Neoporphyra haitanensis	phctrl-1	46845232	40513892	86.48%	9084
	phctrl-2	45846158	39544569	86.25%	9105
	phctrl-3	45400210	38765442	85.39%	9186
	ph6h-1	44259130	38144592	86.18%	9014
	ph6h-2	47994240	41333500	86.12%	9198
	ph6h-3	46263452	39623759	85.65%	8992
	ph1d-1	46346876	40733544	87.89%	9023
	ph1d-2	45698938	38638627	84.55%	9033
	ph1d-3	47308928	41911634	88.59%	8943
	ph2d-1	45588112	40456334	88.74%	9208
	ph2d-2	46955396	41351005	88.06%	9136
	ph2d-3	48609630	42897222	88.25%	9289
	ph3d-1	50046094	43686576	87.29%	9474
	ph3d-2	45900880	39645383	86.37%	9329
	ph3d-3	49125208	42752181	87.03%	9568
	ph5d-1	50138918	42970703	85.70%	9757
	ph5d-2	42741442	36136261	84.55%	9730
	ph5d-3	45323392	38959084	85.96%	9681

Table S2. List of the sporangia-specific genes.

ID	CDD	nr_annotation	FPKM-ctrl	FPKM-6h	FPKM-1d	FPKM-2d	FPKM-3d	FPKM-5d
py00489	Hint	protein Spindly-like	0.01	0.00	0.18	0.00	0.01	10.31
py02444	Hint	unnamed protein product	0.64	0.58	0.17	0.18	0.11	39.51
py03970	Hint	hypothetical protein CHLNCDRAFT_136623	0.60	0.96	4.55	4.22	4.07	147.69
py04275	Hint	desert hedgehog protein precursor	2.25	3.98	3.29	4.19	3.68	110.37
py04656	Hint	protein Spindly-like	0.27	1.49	0.63	0.88	0.58	50.11
py06088	Hint	sonic hedgehog protein	0.17	0.29	0.33	0.44	0.70	14.32
py07906	Hint	unnamed protein product	0.32	0.07	0.02	0.13	0.05	33.76
py02928	ANK	serine/threonine-protein phosphatase 6 regulatory ankyrin repeat subunit B-like	0.40	0.01	0.03	0.05	0.06	14.26
py08932	ANK	serine/threonine-protein phosphatase 6 regulatory	0.19	0.32	0.88	0.70	1.34	15.35
py09772	ANK	ankyrin-3-like isoform X11	0.10	0.01	0.00	0.00	0.05	24.33
py00539	Malectin	unnamed protein product	0.63	0.30	0.88	1.31	1.06	225.61
py02072	Malectin	PKD domain containing protein	0.16	0.49	0.28	0.02	0.06	1044.91
py04245	Malectin	hypothetical protein	0.09	0.05	0.01	0.01	0.02	18.45
py00583	Branch	Xylosyltransferase, family GT14	0.15	0.02	0.00	0.00	0.07	162.19
py01554	Branch	Xylosyltransferase, family GT14	0.02	0.00	0.02	0.00	0.21	62.43
py04051	zf-H2C2_2	unnamed protein product	0.05	0.09	0.02	0.08	0.10	14.33
py04271	zf-H2C2_2	unnamed protein product	0.11	0.01	1.77	0.49	0.51	80.52
py01890	P-loop_ NTPase	conserved hypothetical protein	0.14	0.16	0.81	0.11	0.20	21.66
py11063	P-loop_ NTPase	ARL1-ADP-ribosylation factor	0.57	1.73	1.50	0.67	0.65	25.93
py06072	DUF563	unnamed protein product	0.18	0.03	0.01	0.02	0.00	11.97
py06494	DUF563	transferase, transferring lycosyl groups	0.60	0.31	0.74	1.58	2.89	21.40
py07648	PAN_APPLE	unnamed protein product	0.63	0.02	0.17	0.12	0.08	336.62
py01568	PAN_APPLE	unnamed protein product	1.50	1.56	2.21	2.12	1.89	265.68
pyi00299	PLN02603	unnamed protein product	0.31	0.08	0.01	0.21	0.16	10.25
py11444	Oscp1	protein OSCP1 isoform X2	1.69	0.61	0.58	0.91	1.07	81.46
py11347	SDR_c	3-oxoacyl-(acyl-carrier-prot ein) reductase	0.04	0.14	0.26	0.12	0.30	84.57

py11054	STKc_CAMK	Protein-serine/threonine kinases Rhodoplastic	0.23	0.00	0.00	0.03	0.08	25.01
py10793	ALP_like	2,3-bisphosphoglycerate-independent hosphoglycerate mutase	0.54	0.20	0.16	0.16	0.32	31.89
py10522	Glyco_hydro_1	beta-1,6-glucan synthase	0.33	1.31	3.57	1.25	1.83	121.73
py10047	ATP-synt_D	V-type H+-transporting ATPase subunit d	0.06	0.00	0.08	0.02	0.04	20.79
py08733	IPT	Kelch repeat-containing protein	0.17	0.12	0.15	0.28	0.15	13.45
py08671	zf-MYND	set domain protein	0.24	0.00	0.51	0.05	0.23	31.09
py08647	MFS	tetracycline resistance protein	0.01	0.00	0.00	0.00	0.00	99.35
py08177	Peptidase_M16_M	insulin-degrading enzyme	0.19	0.10	1.06	0.45	0.12	32.64
py07871	DnaJ	chaperone protein dnaJ 10	0.01	0.03	0.08	0.03	0.02	80.18
py07677	YABBY	unnamed protein product	0.18	0.50	0.54	0.24	0.38	41.99
py07666	Homeobox_KN	unnamed protein product	0.22	0.05	0.32	0.22	0.13	52.79
py07616	LRR_RI	LRR repeats and ubiquitin-like domain-containing protein/GTPase	0.17	0.02	0.21	0.09	0.01	22.10
py07397	PRK07003	unnamed protein product	0.04	0.03	0.00	0.01	0.02	248.37
py07380	PBP2_NikA_DppA_OppA_like	unnamed protein product	0.23	0.05	0.01	0.00	0.01	196.79
py07075	PKc_like	Ephrin type-A receptor 1	0.22	0.00	0.00	0.00	0.00	34.19
py06550	p450	cytochrome P450 family 808E-CYP808E1	0.03	0.03	0.01	0.01	0.01	21.43
py05050	NADB_Rossmann	NAD(P)H azoreductase-like	0.48	0.20	0.07	0.08	0.16	31.36
py04965	TTR-52	unnamed protein product	0.21	0.07	0.42	0.07	0.10	936.17
py04531	Translin-like	unnamed protein product	1.11	0.41	0.78	1.12	0.88	42.97
py04317	E1-E2_ATPase	P-type ATPase	0.68	0.09	0.17	0.59	0.24	105.40
py04000	AFD_class_I	AMP-dependent synthetase	0.06	0.07	0.02	0.03	0.02	16.15
py03498	AlgLyase	alginate lyase	0.66	0.19	0.30	0.18	0.26	23.00
py03210	GFO_IDH_MocA	inositol 2-dehydrogenase	0.11	0.01	0.01	0.10	0.03	15.88
py03164	choice_anch_A	unnamed protein product	0.19	0.10	0.00	0.00	0.00	14.10

py01806	AdoMet_MTases	FkbM family methyltransferase	0.47	0.21	0.06	0.02	0.09	16.54
py01550	Exostosin	Glycosyltransferase, family GT47	0.38	0.08	0.04	0.06	0.02	41.93
py01476	Methyltransf_11	cyclopropane fatty acid synthase and related methyltransferase	0.10	0.00	0.05	0.00	0.02	142.08
py00653	SFP1	zinc finger protein 25-like	0.18	0.16	0.21	0.05	0.17	11.29
pyi00601	N/A	unnamed protein product	0.72	0.57	1.90	0.95	1.83	40.56
pyi00396	N/A	unnamed protein product	0.45	0.14	0.16	0.14	0.33	16.77
pyi00320	N/A	unnamed protein product	0.29	0.37	3.80	0.74	0.84	27.92
pyi00226	N/A	unnamed protein product	0.50	0.40	0.20	0.51	0.07	19.93
pyi00056	N/A	unnamed protein product	0.11	0.00	0.00	0.11	0.00	13.22
py11397	N/A	unnamed protein product	0.47	0.76	2.19	2.11	2.19	27.96
py11278	N/A	alpha-1,2-mannosidase	3.77	2.36	2.63	1.99	2.72	270.55
py10527	N/A	unnamed protein product	0.61	0.66	0.68	0.41	0.31	59.03
py10361	N/A	ASPO1527	1.50	1.15	1.86	2.23	2.89	574.90
py10225	N/A	unnamed protein product	0.31	0.47	2.39	0.30	0.22	10.61
py10218	N/A	unnamed protein product	0.19	0.04	0.01	0.06	0.05	19.78
py10193	N/A	unnamed protein product	0.45	0.73	1.01	0.59	0.40	78.53
py09997	N/A	unnamed protein product	0.95	0.81	1.45	1.87	1.20	134.28
py09849	N/A	chitinase	0.48	0.00	0.00	0.17	0.00	20.34
py09834	N/A	unnamed protein product	0.18	0.00	0.23	0.09	0.02	10.30
py09358	N/A	unnamed protein product	0.95	1.56	1.14	0.11	0.41	33.20
py08646	N/A	MFS transporter, tetracycline: hydrogen antiporter	0.69	0.08	0.07	0.07	0.13	215.68
py08607	N/A	unnamed protein product	1.66	0.80	0.95	0.33	0.18	58.13
py08435	N/A	unnamed protein product	0.34	0.06	0.27	0.27	0.51	11.92
py08277	N/A	unnamed protein product	0.71	1.56	1.02	1.48	1.69	42.56
py08112	N/A	ASPO1527	2.18	0.53	0.67	1.11	0.91	2418.66
py07463	N/A	unnamed protein product	0.26	0.84	0.57	0.34	0.60	17.18
py07420	N/A	hypothetical protein	2.13	0.31	0.84	0.92	1.18	275.48
py07220	N/A	unnamed protein product	0.06	0.00	0.04	0.00	0.03	131.01
py06987	N/A	unnamed protein product	1.20	2.65	0.20	0.54	0.22	177.84
py06546	N/A	unnamed protein product	0.22	0.02	0.00	0.04	0.03	11.18
py06196	N/A	unnamed protein product	0.45	0.50	1.91	0.98	0.88	33.56
py06114	N/A	hypothetical protein	0.03	0.29	0.80	0.92	0.45	22.75
py05987	N/A	ASPO2608	0.60	0.03	0.36	0.08	0.02	6566.04
py05848	N/A	unnamed protein product	0.12	0.19	1.80	0.43	0.22	27.58
py05684	N/A	ASPO2608	0.13	0.02	0.25	0.04	0.00	4888.16
py05615	N/A	unnamed protein product	0.38	0.79	2.44	0.78	0.48	24.11

py05016	N/A	unnamed protein product	0.10	0.00	0.09	0.09	0.08	236.91
py05015	N/A	unnamed protein product	1.72	2.67	4.10	3.40	2.94	81.11
py04868	N/A	unnamed protein product	2.03	3.27	3.00	3.22	2.64	71.21
py04687	N/A	unnamed protein product	0.21	1.20	2.56	0.17	0.30	20.92
py04679	N/A	unnamed protein product	0.23	0.05	0.54	0.02	0.21	1249.82
py04554	N/A	ASPO2608	3.73	3.35	2.25	1.56	1.76	2789.60
py04471	N/A	unnamed protein product	1.18	2.00	3.53	1.18	2.36	123.23
py04455	N/A	glycosyltransferase	1.48	0.05	0.10	0.05	0.01	85.40
py04342	N/A	unnamed protein product	0.48	0.48	0.67	0.16	0.28	21.83
py04287	N/A	unnamed protein product	0.20	0.00	1.59	1.03	0.24	105.18
py04234	N/A	unnamed protein product	0.35	0.05	0.28	0.17	0.21	54.89
py04103	N/A	unnamed protein product	0.13	0.04	3.41	0.25	0.17	31.47
py03544	N/A	unnamed protein product	0.09	0.13	1.04	0.34	0.10	14.00
py02798	N/A	unnamed protein product	0.12	0.02	0.07	0.47	0.46	148.06
py01980	N/A	unnamed protein product	0.13	1.45	0.22	0.14	0.27	38.69
py01966	N/A	SnoaL-like domain protein	1.10	1.03	0.59	0.27	0.21	44.53
py01919	N/A	unnamed protein product	0.31	0.01	0.11	0.06	0.03	12.70
py01907	N/A	ASPO1527	0.01	0.09	0.17	0.10	0.03	76.92
py01906	N/A	ASPO1527	0.01	0.03	0.05	0.02	0.02	89.18
py01586	N/A	unnamed protein product	0.79	2.28	3.11	3.00	2.44	794.30
py00848	N/A	unnamed protein product	0.77	0.41	0.39	0.37	0.25	788.34
py00622	N/A	tetratricopeptide TPR_2	0.20	0.01	0.00	0.01	0.02	11.89

Table S3. Primer sequences used in qRT-PCR.

Gene name	Gene ID	Forward primer	Reverse primer
UBC	py04835	5' CGCTGACCGTTTCCAAG 3'	5' CGACTGCGGTTGGACTT 3'
RBOH-1	py00905	5' CGACCGCACCCACACCGAG 3'	5' CCCGCCACCAGGTCATGCAG 3'
RBOH-2	py04018	5' CGCCTCTTCCCCTGGTCG 3'	5' AAAGACCATGTGCGCCTCGT 3'
RBOH-3	py02900	5' CGCCGCTTCTCCTCTACACCT 3'	5' CCGCAGCACGTCCGAGTCAC 3'
SOD	py09809	5' TCAAGGCGACGCTGAACAAC 3'	5' CAACGTGCGGCAGGGAGAT 3'
GLR	py01585	5' GTATCCTTCCGACCGCCCTGT 3'	5' GCCTCAGCAACCCGGTTCCAC 3'
calmodulin	py01734	5' CCTCACCCTCATGTCACGCAAG 3'	5' CCCGGATCATCTCGCTCACC 3'
CDPK-1	py05275	5' ATTGTCCTCCAGTCGCAGGT 3'	5' GAAGCGGCCCTTGCACACCA 3'
CDPK-2	py08655	5' TGTGGCCTGTCTACACGACCAT 3'	5' TACGTCTCGCCACGGATGACC 3'
PCNA	py06123	5' ACCGCATCACCACCTTTGAGC 3'	5' AGGTTCCCCTTGCCAATGTCG 3'
MSH2	py02837	5' AAGCTCATCCGCGCCAAGTCC 3'	5' ACTCGTACTCGGCCATCAGGT 3'
MSH6	py05915	5' TCTTCCACCGCCCGAAAGAGC 3'	5' CGTTCCACCTCGGGAGTCCA 3'
galactosidase 1	py08621	5' TCATTGCGCGTATGGACCACT 3'	5' CGCAAGGTCCAGCCACTCGTC 3'
cellulase-1	py05706	5' CGAGACCGCCCTTGTCCAC 3'	5' CGTCTCCATTGTACGCAGTGACC 3'
cellulase-2	py11230	5' CAGTGGCGTGTATGGGGA 3'	5' CGGTCGGGCTGGTTGA 3'
expansin	py09765	5' GGCTGGGGCGATTCTGAC 3'	5' CCACCGCAAACACAAGGTATT 3'

Dataset 1. The list of DEGs in *N. yezoensis* in the 6h, 1d, 2d, 3d and 5d samples. The pre-wound sample was used as control. Average FPKM values and padj values at all the time points were presented for each DEG. DEGs of each time point were listed in an individual excel sheet.

Dataset 2. The list of TFs exhibiting differential transcription in 6h, 1d, 2d, 3d and 5d samples in *N. yezoensis*. The pre-wound sample was used as control. Average FPKM values at all the time points were presented for each TF. TFs of each time point were listed in an individual excel sheet.

Fig. S1. Venn diagram(A) showing the DEGs identified at each time point in *N. yezoensis*, and *PCA* analysis(B) of sample after wounding in *N. yezoensis*.

Fig. S2. Biological functions encoded by DEGs shared by the five time points. A, Functional enrichment of shared DEGs in Gene Ontology (GO) categories. B, Functional enrichment of shared DEGs in the Kyoto Encyclopedia of Genes and Genome (KEGG) pathways.

Fig. S3. Venn diagram showing the differentially expressed TFs identified at each time point in *N. yezoensis*.

Fig. S4. Sample clustering of transcriptome data in *N. haitanensis*. The heatmap illustrates HCL clustering of all DEGs at the five time points in *N. haitanensis.* The pre-wound control, hour 6 and day 1 each formed distinct clusters. Ph3d-2 were separated from ph3d-1 and ph3d-3, and mixed with ph-2d samples. Moreover, samples of the two time points were clustered with ph5d samples, suggesting the similarity in global transcriptional patterns in ph2d, ph3d and ph5d samples.

Fig. S5. Transcriptional variations of photosynthesis-related genes in the two species. A, Transcriptional variations of photosynthesis related genes in *N. yezoensis*. The blue line represent values of each gene and the red line represents mean values. B, Transcriptional variations of photosynthesis related genes in *N. haitanensis*. The blue line represent values of each gene and the red line represents mean values.

Fig. S6. Transcriptional variation of *RBOH*s, *SOD*s, *AQP*s. A, Phylogeny of *Neopyropia RBOH* genes constructed in MEGA5 through the Maximal Likelihood (ML) method. Numerical value of each branch in the tree represents genetic distance; Node values represent the test confidence (bootstrap=1000). B, A heatmap illustrating the transcriptional dynamics of *RBOH* genes in response to wound stress in *N. yezoensis* and *N. haitanensis*.. Transcriptional variation was indicated by the $\log_2$ value of foldchange in FPKM comparing to intact thalli. C, Phylogeny of *Neopyropia SOD* genes. D, A heatmap illustrating the transcriptional dynamics of *SOD* genes in response to wound stress in *N. yezoensis* and *N. haitanensis*. E, Phylogeny of *Neopyropia AQP* genes. F, A heatmap illustrating the transcriptional dynamics of

AQP genes in response to wound stress in *N. yezoensis* and *N. haitanensis*. (Genes with FPKM < 5 were not shown in the heatmap).

Fig. S7. Transcript dynamics of selected *N. yezoensis* genes related to this study as measured by qRT-PCR. They are *RBOH*s (A-C), *SOD* (D), *GLR* (E), *CAM* (F), *CDPK*s (G-H), *PCNA* (I), *MSH*s (J-K), *galactosidase* (L), *cellulases* (M-N), *expansin* (O). Each data point represents the average of three biological replicates. Each sample was analyzed in technical triplicates. Values represent means ± SD (n=3).

Fig. S8. Phylogeny of *Neopyropia CDPK* genes. The Phylogenetic tree was constructed in MEGA5 through the ML method with default parameters. The *CDTKL* genes from *P. umbilicalis* were used as outliers. The orthologous relationships of *N. yezoensis* and *N. haitanensis* genes were deduced based on this tree.

Fig. S9. Transcriptional variation of *N. haitanensis CDPK*s. The heatmap shows the $\log_2$ values of FPKM foldchanges at each time point compared to the intact sample. Compared to the transcriptional patterns of *N. yezoensis CDPK* genes revealed in the heatmap in fig. 3H, *N. haitanensis CDPKs* generally exhibited down-regulated transcription.

Fig. S10. Transcription variation of mismatch repair and base excision repair related genes. A, Transcriptional variations of mismatch repair and base excision repair related genes in *N. yezoensis*. The red line represents the mean values of $\log_2$ (foldchange) of all the related genes, and the error bars indicate standard errors. B, Transcriptional variations of mismatch repair and base excision repair related genes in *N. haitanensis.*

Fig. S11. Morphological changes *N. haitanensis* cut fragments with exogenous rapamycin addition. Same concentration of rapamycin was added to *N. haitanensis* fragments. The time points after cutting were presented at the up-left corners of each panel. On day 5, the marginal region of the fragment was repaired and cells remains alive.

Fig. S12. Phylogeny of *cyclin* and *CDK* genes. The phylogenetic tree was constructed as described in Fig. S4.

Fig. S13. Transcriptional variation of *GH* genes. Mannosidase and galactosidase genes were indicated with hollow and solid triangles respectively.

Fig. S14. Functional enrichment of sporangia-specific genes in GO categories.

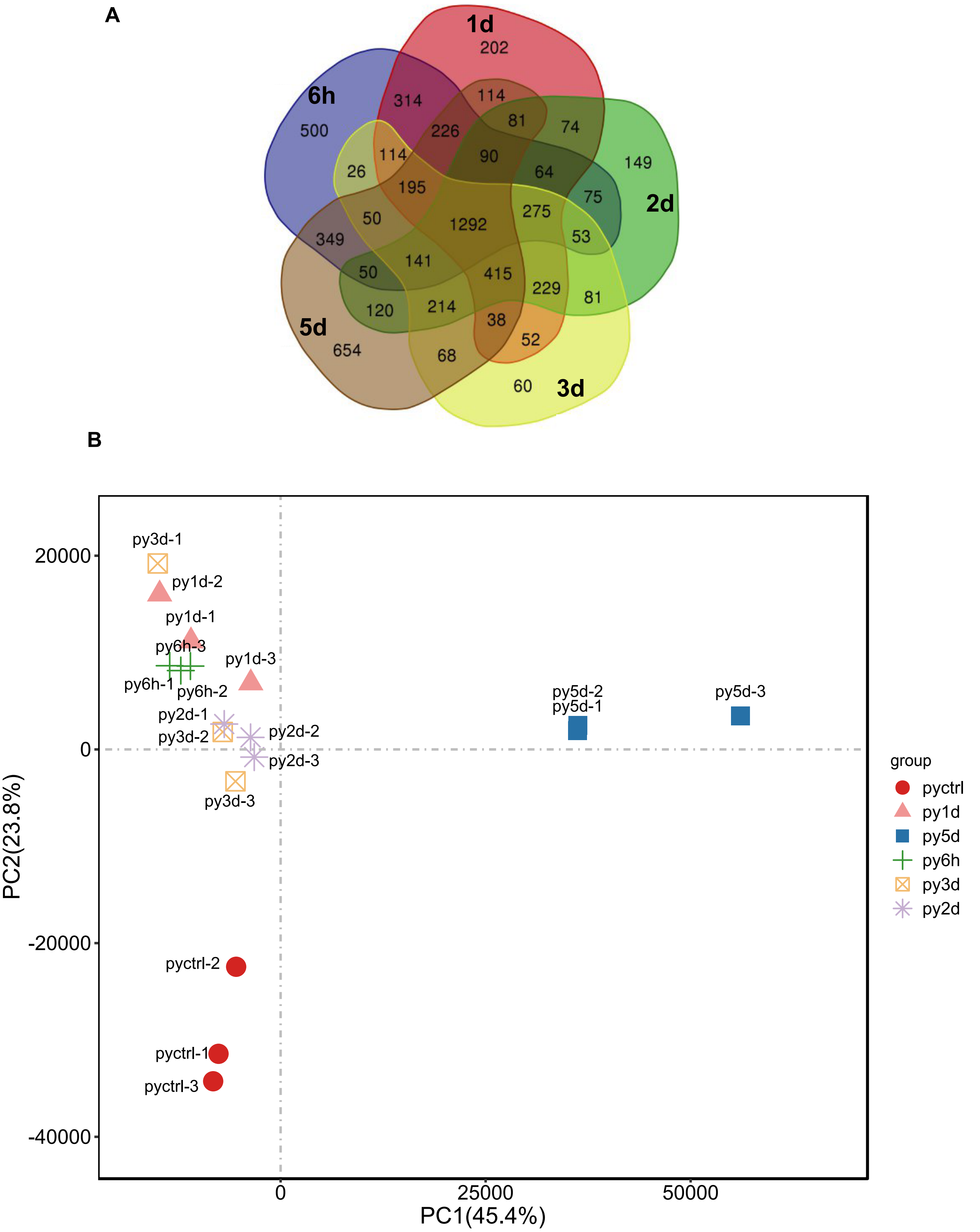

Fig. S1. Venn diagram(A) showing the DEGs identified at each time point in *N. yezoensis*, and *PCA* analysis(B) of sample after wounding in *N. yezoensis*.

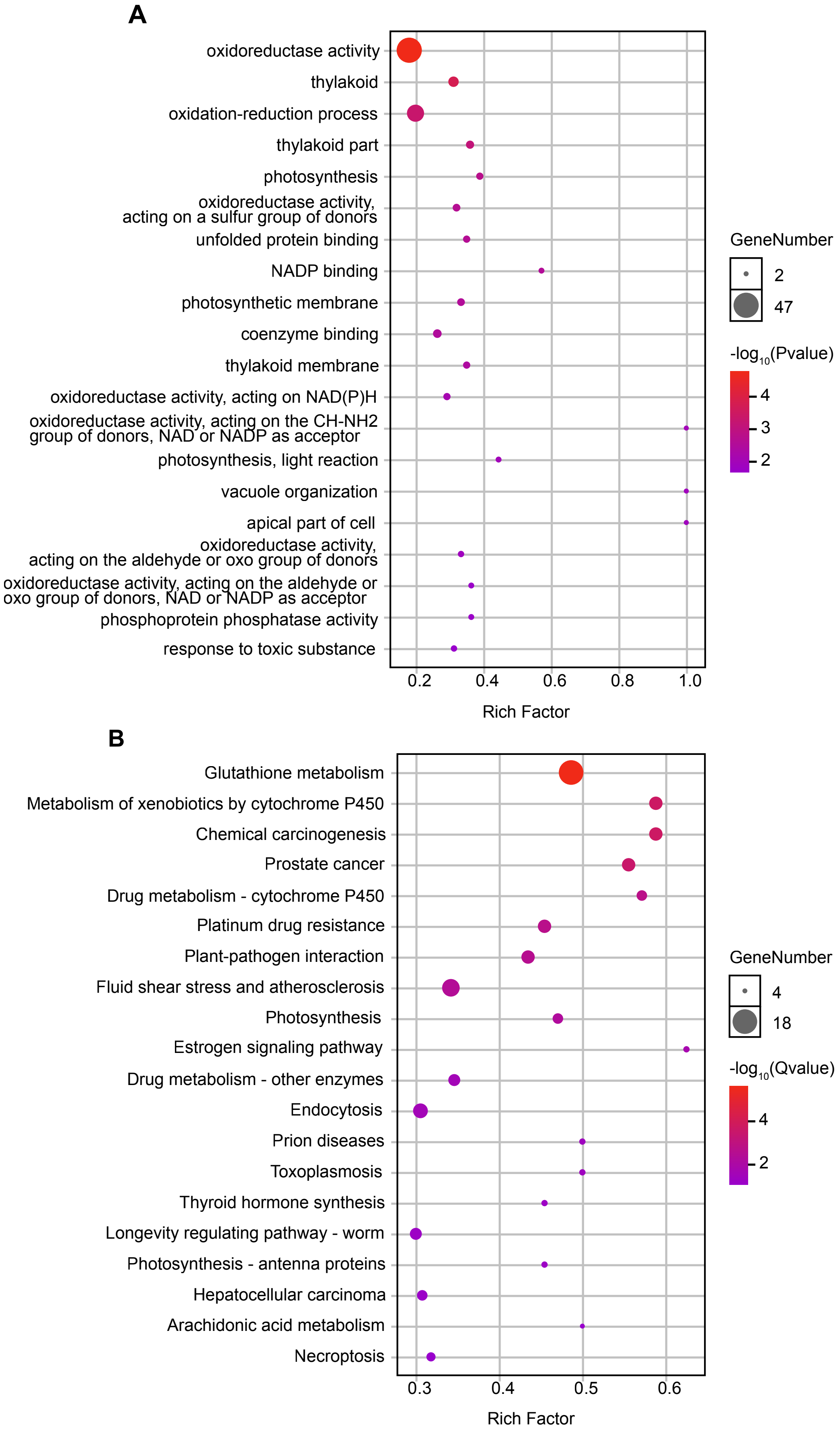

Fig. S2. Biological functions encoded by DEGs shared by the five time points.
A, Functional enrichment of shared DEGs in Gene Ontology (GO) categories.
B, Functional enrichment of shared DEGs in the Kyoto Encyclopedia of Genes and Genome (KEGG) pathways.

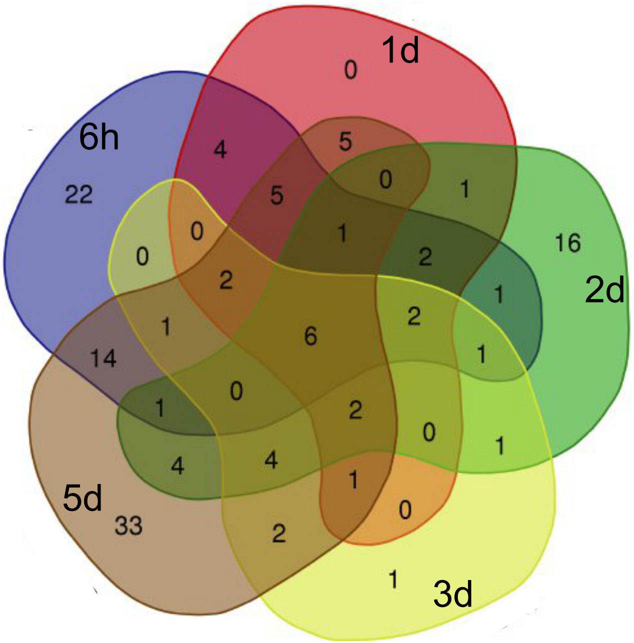

Fig. S3. Venn diagram showing the differentially expressed TFs identified at each time point in *N. yezoensis.*

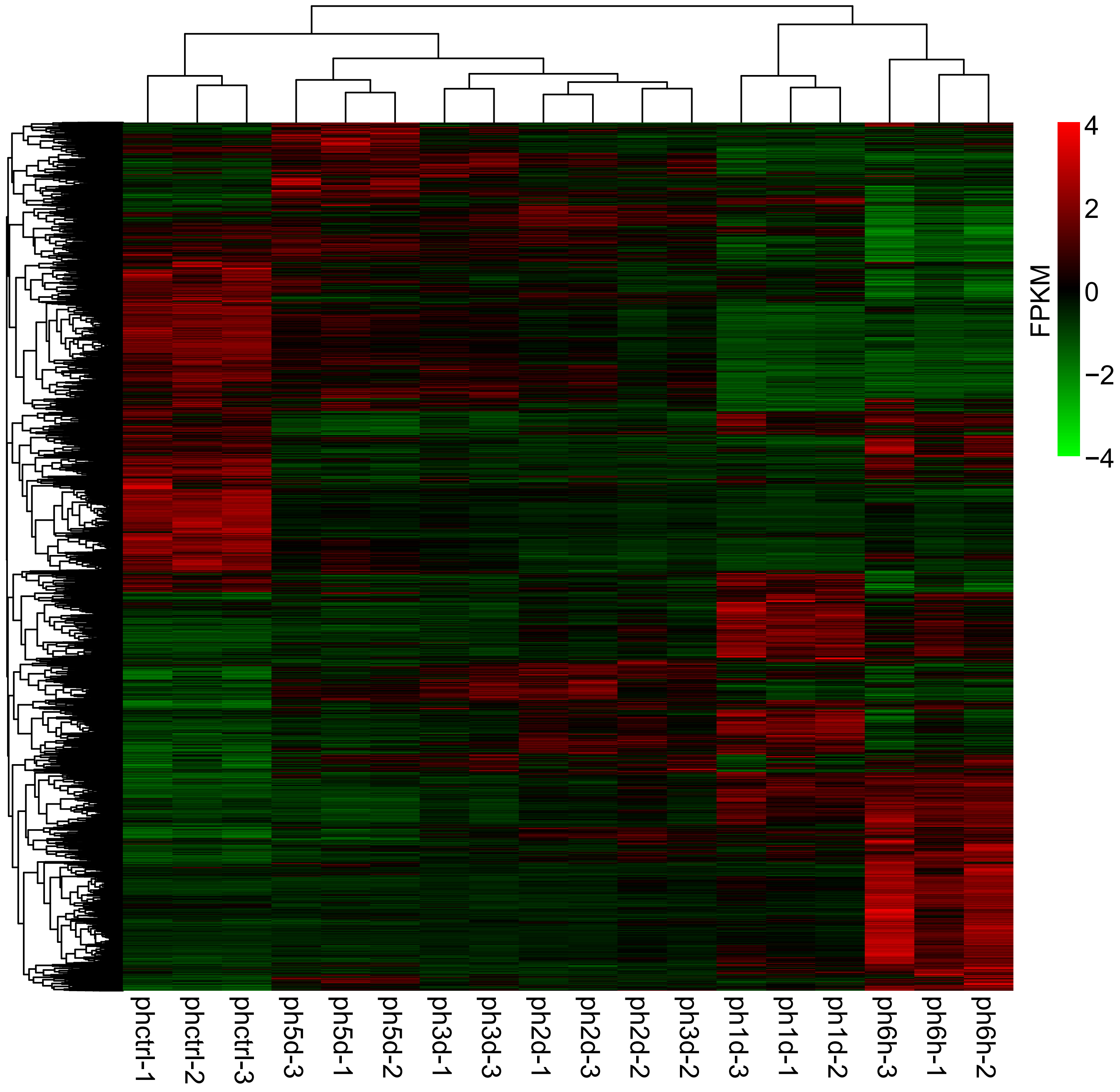

Fig. S4. Sample clustering of transcriptome data in *N. haitanensis*. The heatmap illustrates HCL clustering of all DEGs at the five time points in *N. haitanensis*. The pre-wound control, hour 6 and day 1 each formed distinct clusters. Ph3d-2 were separated from ph3d-1 and ph3d-3, and mixed with ph-2d samples. Moreover, samples of the two time points were clustered with ph5d samples, suggesting the similarity in global transcriptional patterns in ph2d, ph3d and ph5d samples.

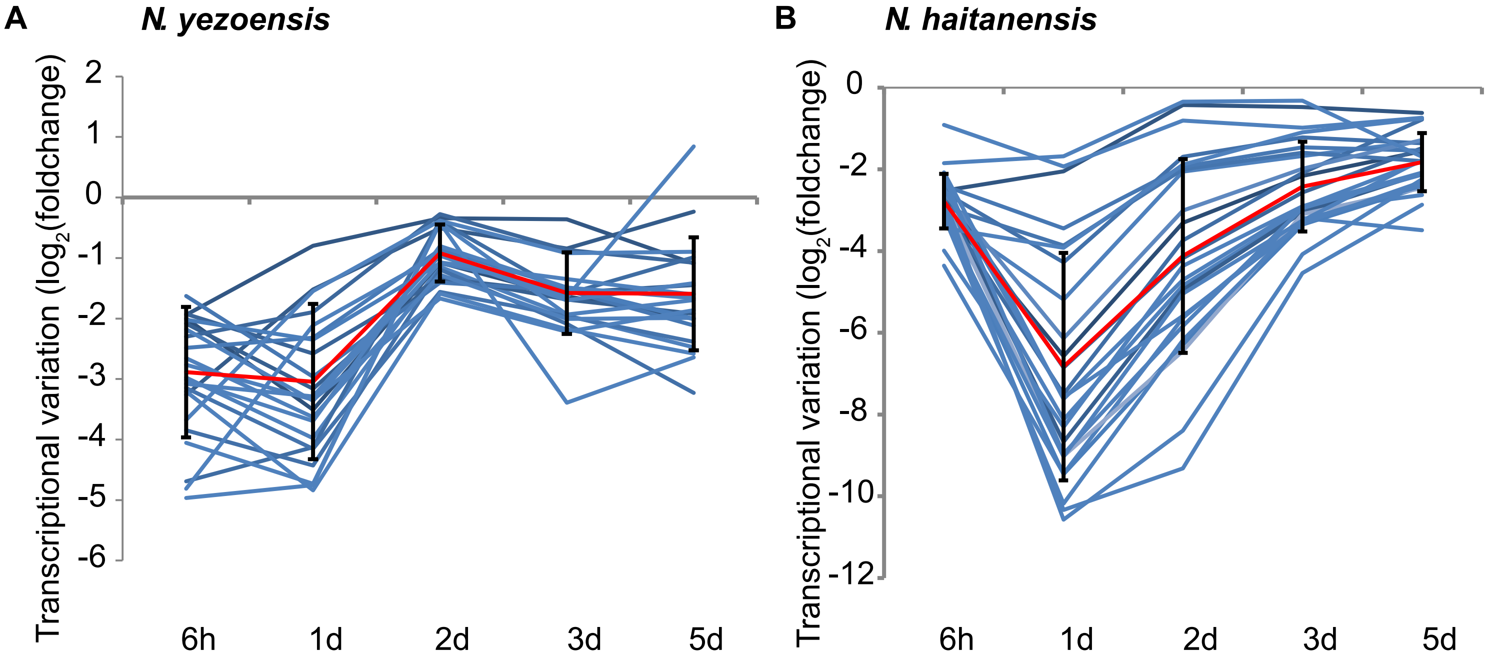

Fig. S5. Transcriptional variations of photosynthesis-related genes in the two species.

A, Transcriptional variations of photosynthesis related genes in *N. yezoensis.*

The blue line represent values of each gene and the red line represents mean values.

B, Transcriptional variations of photosynthesis related genes in *N. haitanensis.*

The blue line represent values of each gene and the red line represents mean values.

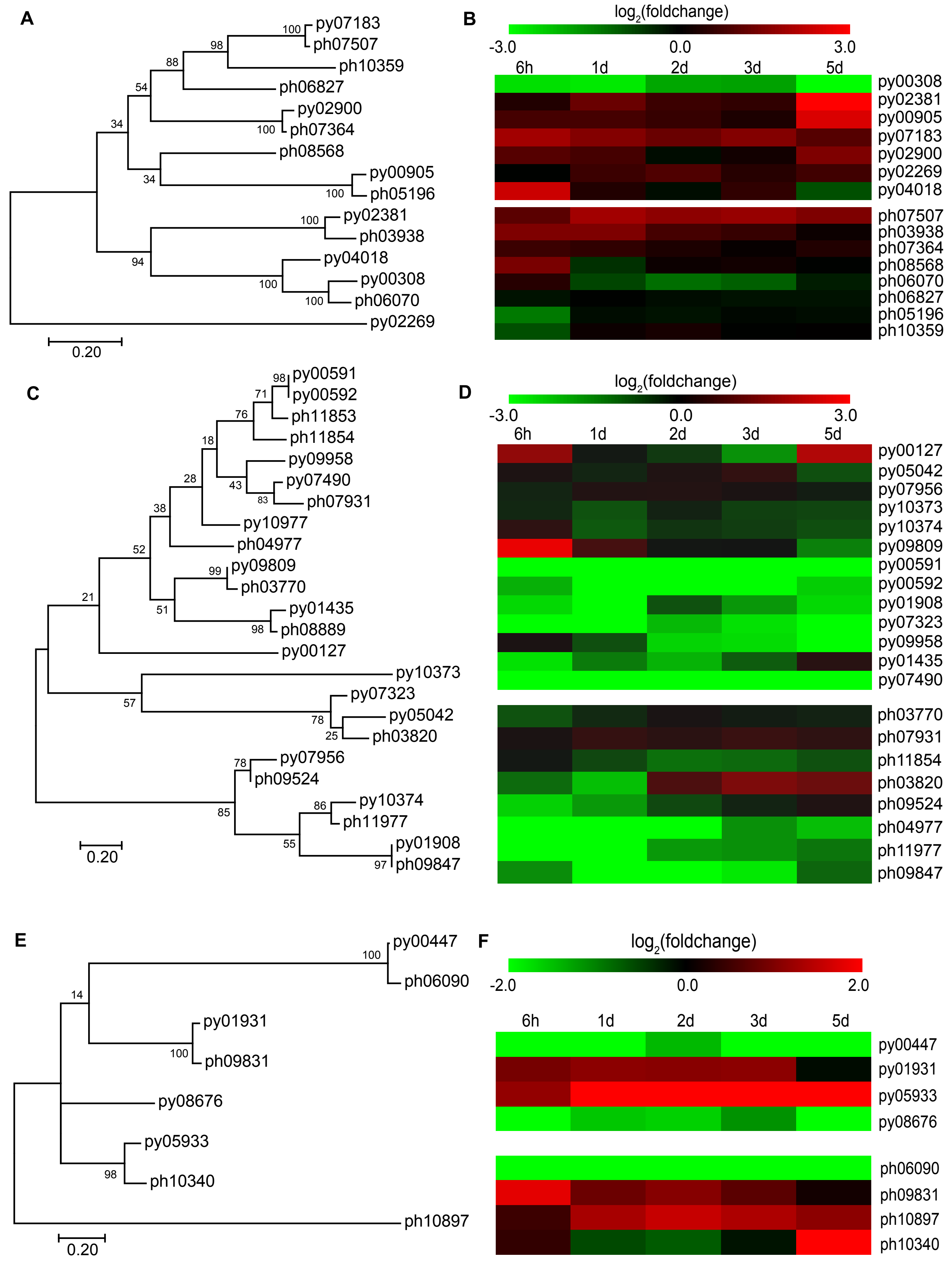

Fig. S6. Transcriptional variation of *RBOH, SOD, AQPs.* A, Phylogeny of *Neopyropia RBOH* genes constructed in MEGA5 through the Maximal Likelihood (ML) method. Numerical value of each branch in the tree represents genetic distance; Node values represent the test confidence (bootstrap=1000). B, A heatmap illustrating the transcriptional dynamics of *RBOH* genes in response to wound stress in *N. yezoensis* and *N. haitanensis*. Transcriptional variation was indicated by the log_2 value of foldchange in FPKM comparing to intact thalli. C, Phylogeny of *Neopyropia SOD* genes. D, A heatmap illustrating the transcriptional dynamics of *SOD* genes in response to wound stress in *N. yezoensis* and *N. haitanensis*. E, Phylogeny of *Neopyropia AQP* genes. F, A heatmap illustrating the transcriptional dynamics of *AQP* genes in response to wound stress in *N. yezoensis* and *N. haitanensis*. (Genes with FPKM < 5 were not shown in the heatmap).

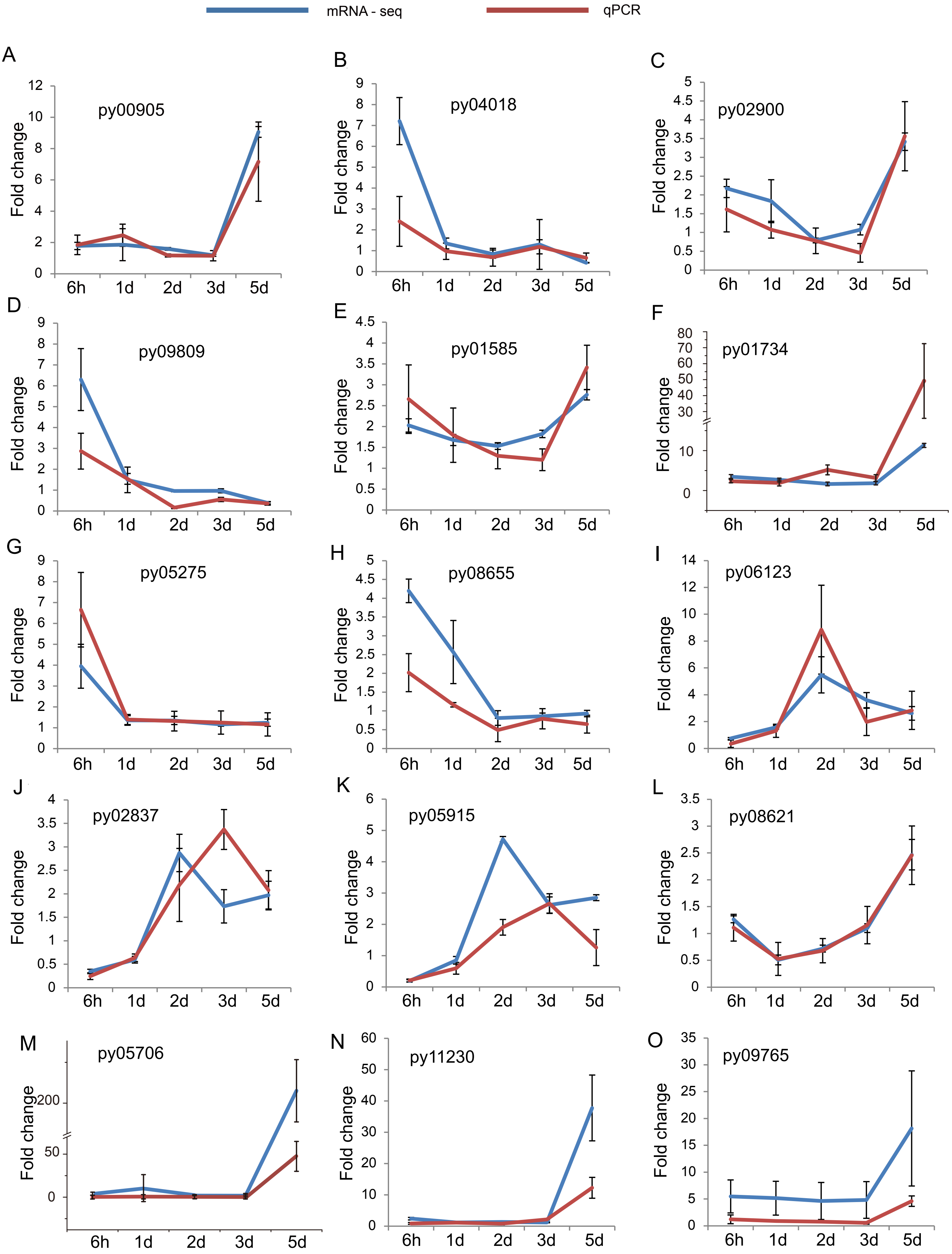

Fig. S7. Transcript dynamics of selected *N. yezoensis* genes related to this study as measured by qRT-PCR. They are *RBOHs* (A-C), *SOD* (D), *GLR* (E), *CAM* (F), *CDPKs* (G-H), *PCNA* (I), *MSHs* (J-K), *galactosidase* (L), *cellulases* (M-N), *expansin* (O). Each data point represents the average of three biological replicates. Each sample was analyzed in technical triplicates. Values represent means ± SD (n=3).

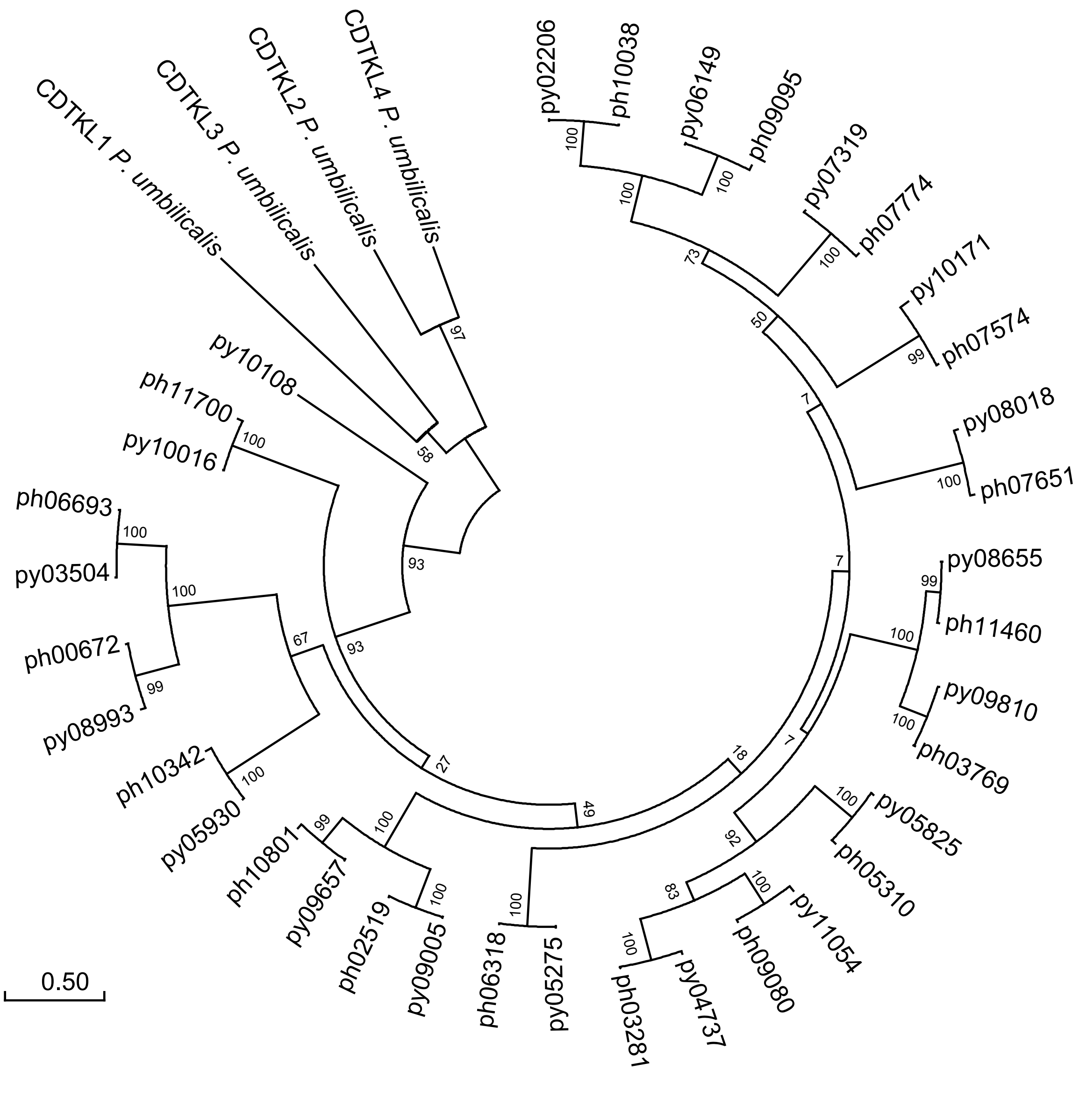

Fig. S8. Phylogeny of *Neopyropia CDPK* genes. The Phylogenetic tree was constructed in MEGA5 through the ML method with default parameters. The *CDTKL* genes from *P. umbilicalis* were used as outliers. The orthologous relationships of *N. yezoensis* and *N. haitanensis* genes were deduced based on this tree.

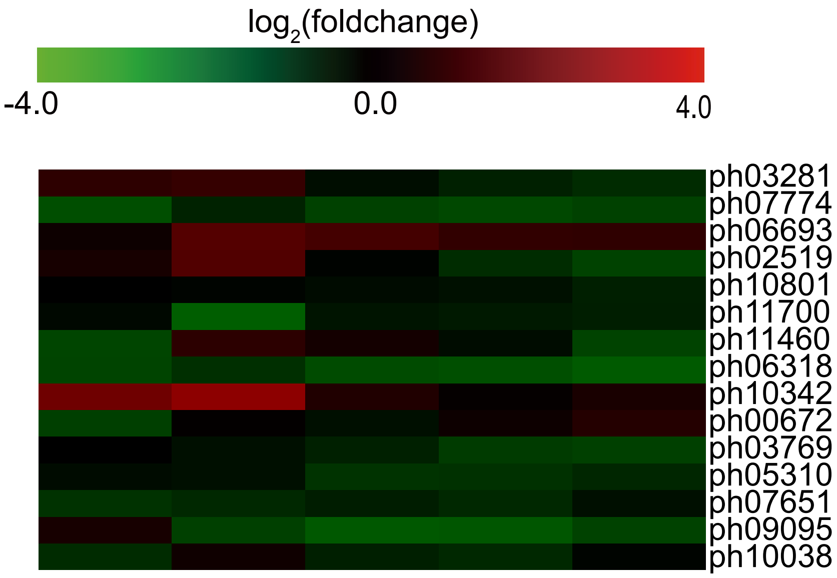

Fig. S9. Transcriptional variation of *N. haitanensis CDPK*s. The heatmap shows the log2 values of FPKM foldchanges at each time point compared to the intact sample. Compared to the transcriptional patterns of *N. yezoensis CDPK* genes revealed in the heatmap in fig. 3H, *N. haitanensis CDPK*s generally exhibited down-regulated transcription.

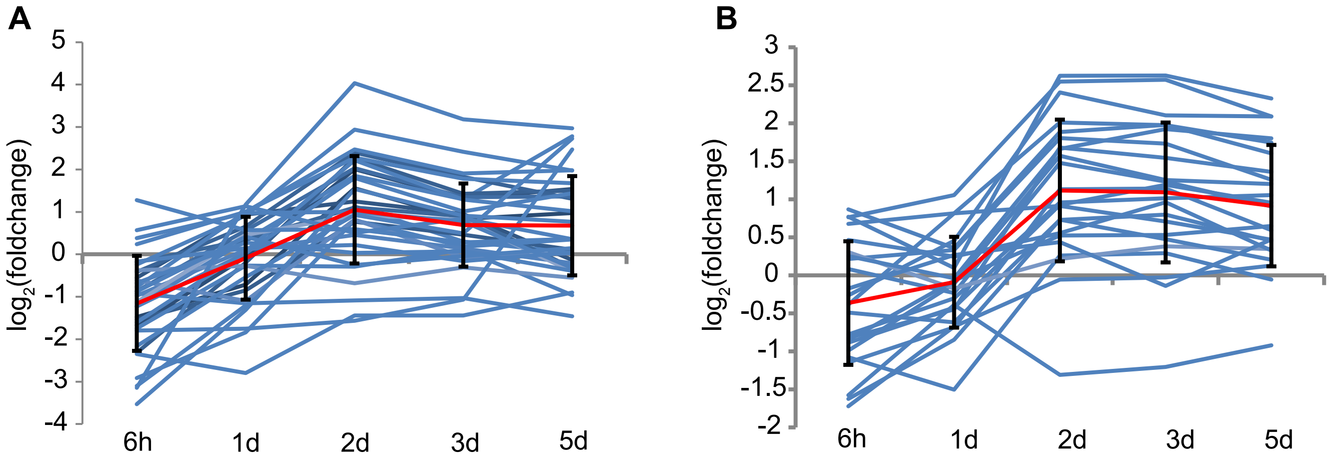

Fig. S10. Transcription variation of mismatch repair and base excision repair related genes. A, Transcriptional variations of mismatch repair and base excision repair related genes in *N. yezoensis*. The red line represents the mean values of $\log_2$(foldchange) of all the related genes, and the error bars indicate standard errors. B, Transcriptional variations of mismatch repair and base excision repair related genes in *N. haitanensis.*

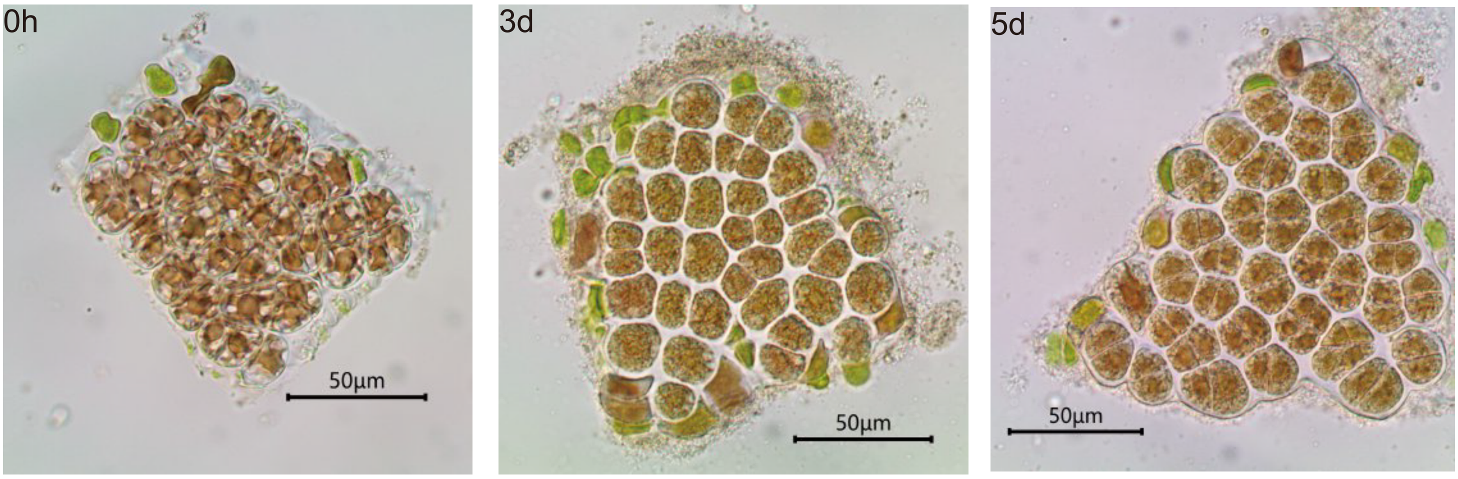

Fig. S11. Morphological changes *N. haitanensis* cut fragments with exogenous rapamycin addition. Same concentration of rapamycin was added to *N. haitanensis* fragments. The time points after cutting were presented at the up-left corners of each panel. On day 5, the marginal region of the fragment was repaired and cells remains alive.

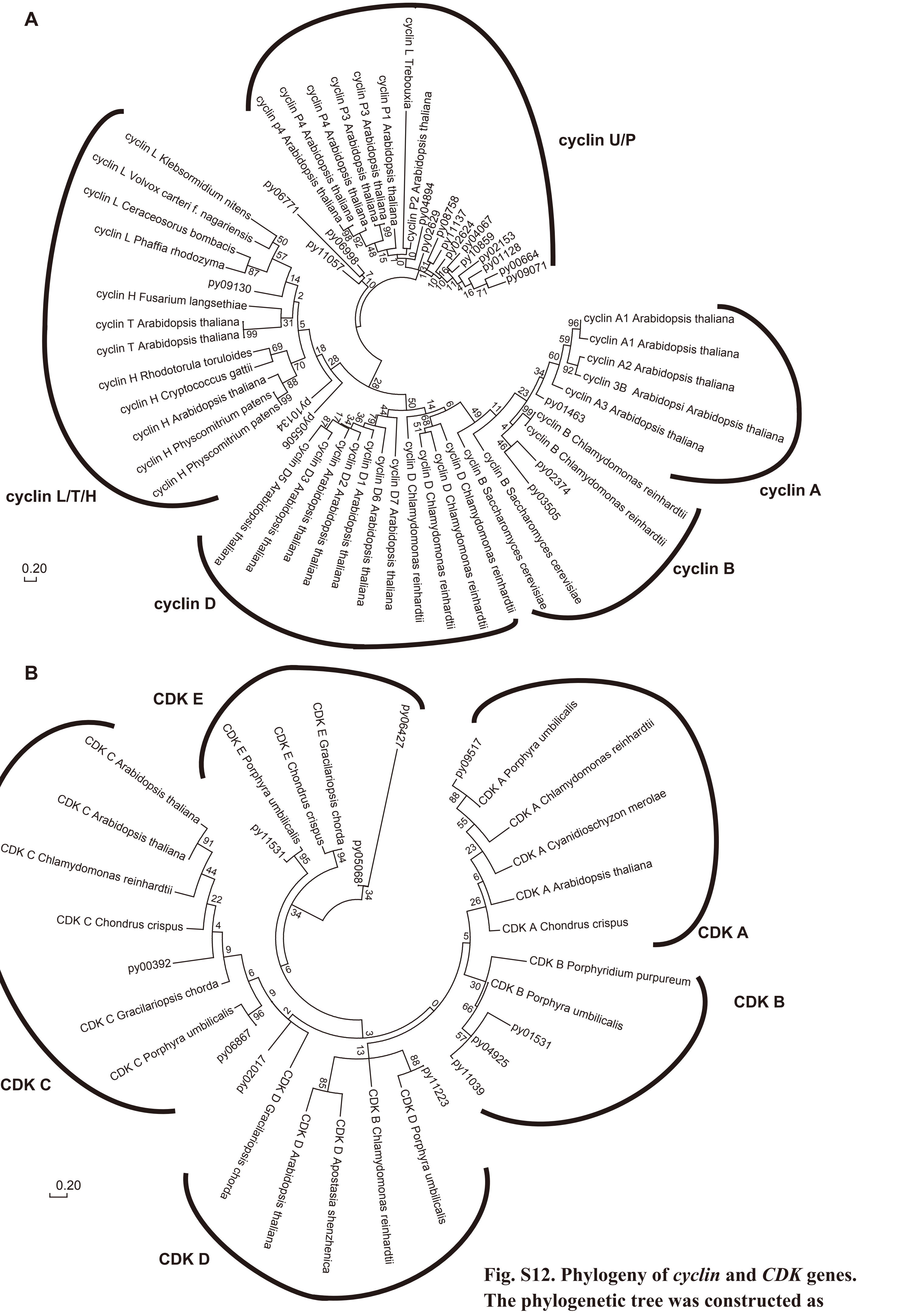

Fig. S12. Phylogeny of *cyclin* and *CDK* genes. The phylogenetic tree was constructed as described in Fig. S4.

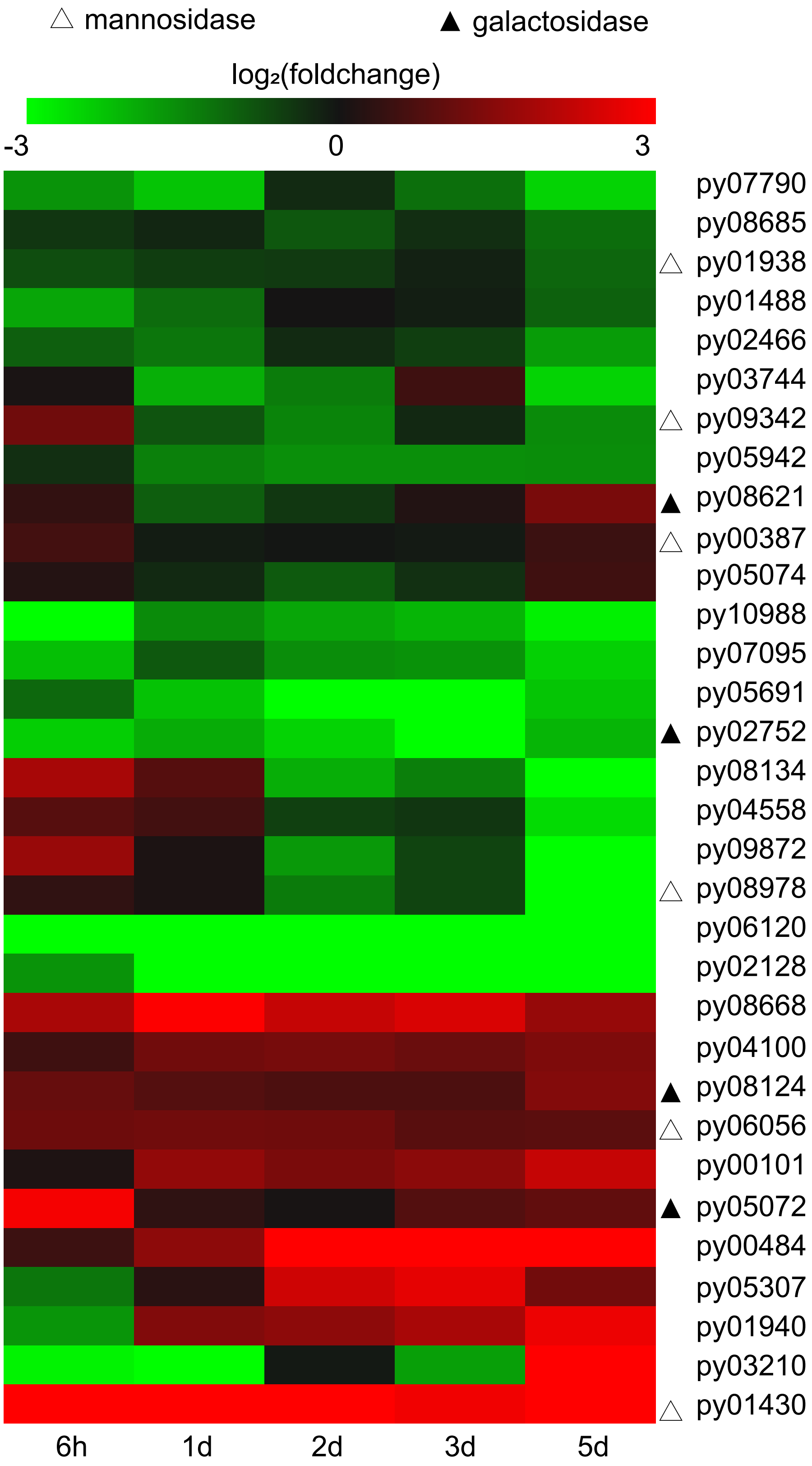

Fig. S13. Transcriptional variation of *GH* genes. Mannosidase and galactosidase genes were indicated with hollow and solid triangles respectively.

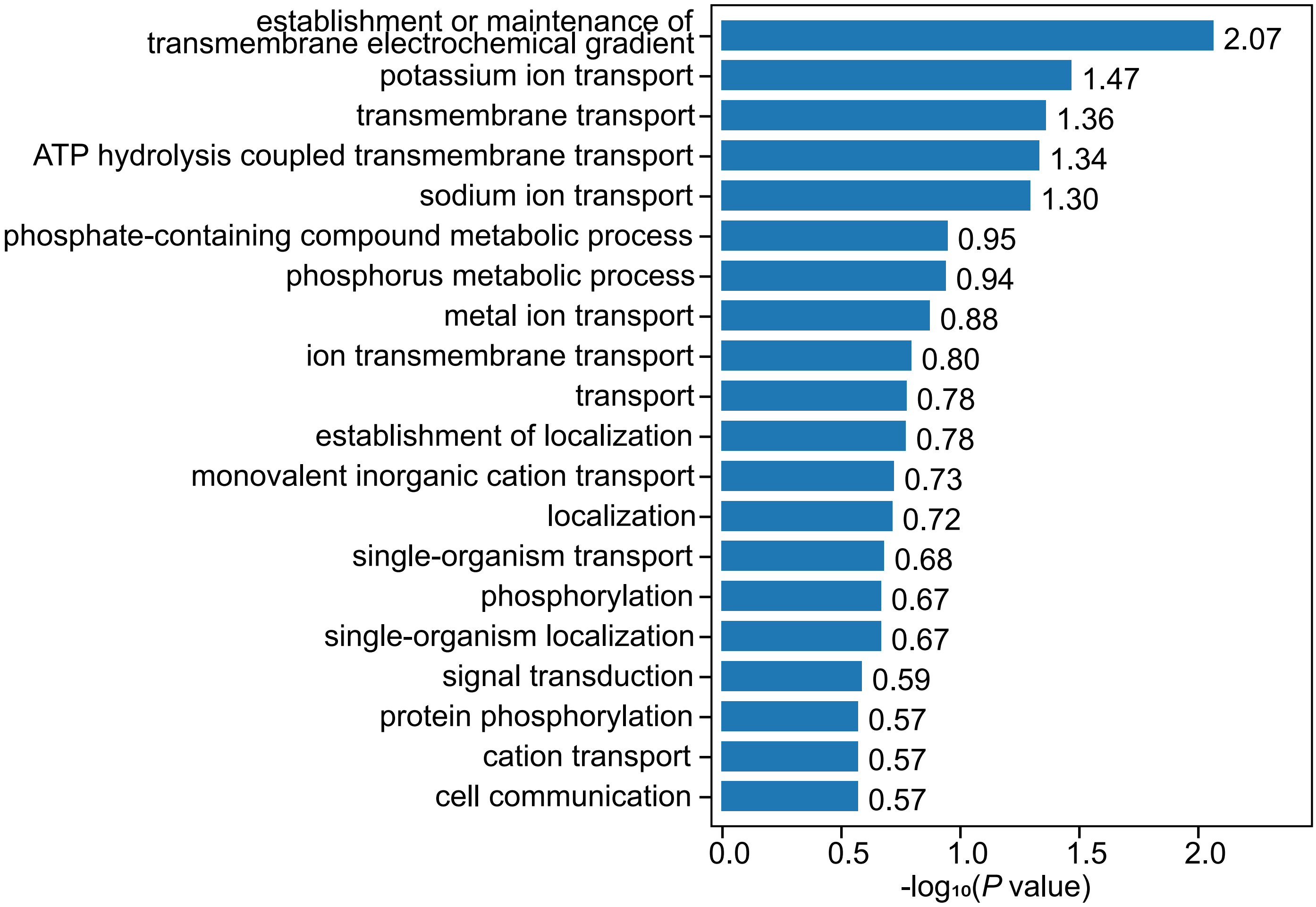

Fig. S14. Functional enrichment of sporangia-specific genes in GO categories.
